# Metabolic Flexibility as an Adaptation to Energy Resources and Requirements in Health and Disease

**DOI:** 10.1210/er.2017-00211

**Published:** 2018-04-24

**Authors:** Reuben L Smith, Maarten R Soeters, Rob C I Wüst, Riekelt H Houtkooper

**Affiliations:** 1 Laboratory of Genetic Metabolic Diseases, Academic Medical Center, Amsterdam, Netherlands; 2 Amsterdam Gastroenterology and Metabolism, Academic Medical Center, Amsterdam, Netherlands; 3 Department of Endocrinology and Metabolism, Internal Medicine, Academic Medical Center, Amsterdam, Netherlands; 4 Amsterdam Cardiovascular Sciences, Academic Medical Center, Amsterdam, Netherlands; 5 Amsterdam Movement Sciences, Academic Medical Center, Amsterdam, Netherlands

## Abstract

The ability to efficiently adapt metabolism by substrate sensing, trafficking, storage, and utilization, dependent on availability and requirement, is known as metabolic flexibility. In this review, we discuss the breadth and depth of metabolic flexibility and its impact on health and disease. Metabolic flexibility is essential to maintain energy homeostasis in times of either caloric excess or caloric restriction, and in times of either low or high energy demand, such as during exercise. The liver, adipose tissue, and muscle govern systemic metabolic flexibility and manage nutrient sensing, uptake, transport, storage, and expenditure by communication via endocrine cues. At a molecular level, metabolic flexibility relies on the configuration of metabolic pathways, which are regulated by key metabolic enzymes and transcription factors, many of which interact closely with the mitochondria. Disrupted metabolic flexibility, or metabolic inflexibility, however, is associated with many pathological conditions including metabolic syndrome, type 2 diabetes mellitus, and cancer. Multiple factors such as dietary composition and feeding frequency, exercise training, and use of pharmacological compounds, influence metabolic flexibility and will be discussed here. Last, we outline important advances in metabolic flexibility research and discuss medical horizons and translational aspects.

## Teaser

Authors review the ways in which metabolic flexibility influences health and disease. The role of major metabolic organs, endocrine signals, key molecular pathways, and their players are discussed.

**Essential Points**

- Metabolic flexibility describes efficient switches in metabolism depending on environmental demand
- Mitochondria play a crucial role in determining metabolic flexibility
- Metabolic inflexibility is a hallmark of many age-related metabolic diseases but also plays a central role in, for instance, cancer and immune metabolism
- Molecular and signaling pathways drive metabolic flexibility and often serve as metabolic sensors
- Metabolic flexibility pathways are therapeutic targets for age-related diseases, similar to caloric restriction or exercise

Human physiology evolved during times of dramatic fluctuations in energy supply and demand. Coping with these changes has entrained the human body with the ability to manage energy metabolism for optimal substrate storage and use during states of either food surplus or famine, and periods of either rest or increased energy demand. The ability to efficiently adapt metabolism depending on demand or supply is known as *metabolic flexibility* (1). In general, the human body can aptly use moderate amounts of carbohydrates, fatty acids, and amino acids. The modern era, however, is characterized by unprecedented levels of food supply (2). This near continuous intake of calorically dense processed foods, combined with physical inactivity, reduces a predilection for, and directly impedes, metabolic flexibility (3). This is caused by substrate competition and metabolic insensitivity, characterized by distorted nutrient sensing, blunted substrate switching, and impaired energy homeostasis (4). Importantly, this metabolic inflexibility may underlie the epidemic changes in metabolic disease that affect all demographic groups and burden health-care systems (5, 6).

## Defining Metabolic Flexibility

Maintaining energy homeostasis requires substrate sensing, trafficking, storage, and utilization, dependent on substrate availability (push concept) and energy requirement (pull concept). Metabolic plasticity (or adaptability) was recognized in 1983 by Saltin and Gollnick (7) when they reviewed the metabolic adaptations of skeletal muscle to exercise (see “Physical exercise”). The term metabolic flexibility was coined by Kelley *et al.* (8) in 1999 when they studied fuel selection in skeletal muscle in lean and obese individuals after an overnight fast. Specifically, they discovered that skeletal muscle of lean individuals showed a remarkable ability to adapt fuel preference to fasting and insulin infusions and were therefore designated as metabolically flexible (8). Insulin-resistant obese patients however manifested a lesser reliance on fatty acid oxidation compared with lean individuals and did not show increased fatty acid oxidation after fasting or reduced fatty acid oxidation after insulin infusion. Because of their inadequate responses to metabolic challenges, these patients were named “metabolically inflexible” (9). More recent work showed that, upon consumption of a high-fat diet, lean subjects with adequate metabolic flexibility were able to increase fatty acid oxidation (FAO) at the expense of glucose, whereas obese individuals were not (10). Lean individuals also showed an increased expression of genes involved in fatty acid transport and oxidation compared with little or no change in their obese counterparts (10).

The concept of metabolic flexibility was particularly linked to the capacity of mitochondria to select fuel in response to nutritional changes (4) and placed mitochondrial function at its core (11). Later, metabolic flexibility quickly expanded to encompass the ability of a given system (whole-body, organ, single cell, or organelle) to handle specific nutrients. At a molecular level, metabolic flexibility relies on the configuration of metabolic pathways that manage nutrient sensing, uptake, transport, storage, and utilization. This metabolic organization is mediated by synthesis, degradation, or activity regulation of key proteins in metabolic circuits or enzymes with a high metabolic flux (11, 12). Metabolic flexibility is not an “on-off” phenomenon, but involves tightly regulated subtle adjustments. Taken together, metabolic flexibility can be understood as an adaptive response of an organism’s metabolism to maintain energy homeostasis by matching fuel availability and demand to periodic fasting, varying meal composition, physical activity, and environmental fluctuations (13, 14).

Here, we review the impact of metabolic flexibility on health and disease. First, we recapitulate how metabolic flexibility is regulated during natural fluctuations in dietary fuel availability and during states of increased demand, such as upon physical exertion. We focus on the mechanistic underpinnings of metabolic flexibility, denote its regulators, and bring the mitochondria forward as central to this process. Second, we examine impaired metabolic flexibility as a cause of diseases such as type 2 diabetes mellitus (T2DM), obesity, cancer, and during conditions of systemic inflammation such as sepsis. Third, we explore how metabolic inflexibility can be reversed by lifestyle or pharmacological interventions. Last, we outline future perspectives for the research field and discuss translational aspects and medical horizons.

## Physiological Relevance of Metabolic Flexibility

### Feeding/fasting

In healthy individuals, metabolism is distinctly different during a state of fasting compared with a state of caloric availability. In the beginning of the 1900s, Francis Benedict described the adaptation to starvation in a single human subject (15). To date, this is the most elaborate and detailed human work on what, in retrospect, can be addressed as metabolic flexibility. Quite some time later, multiple studies quantified reciprocal changes in the adaptation to fasting [reviewed in Soeters *et al.* (16)] emphasizing the dynamic changes in substrate utilization during feeding and fasting. At the same time, many studies investigated nutrient intake, but it was not until the 1970s that a study investigated the postprandial response in relation to glucose and lipids (17).

**Figure 1.**
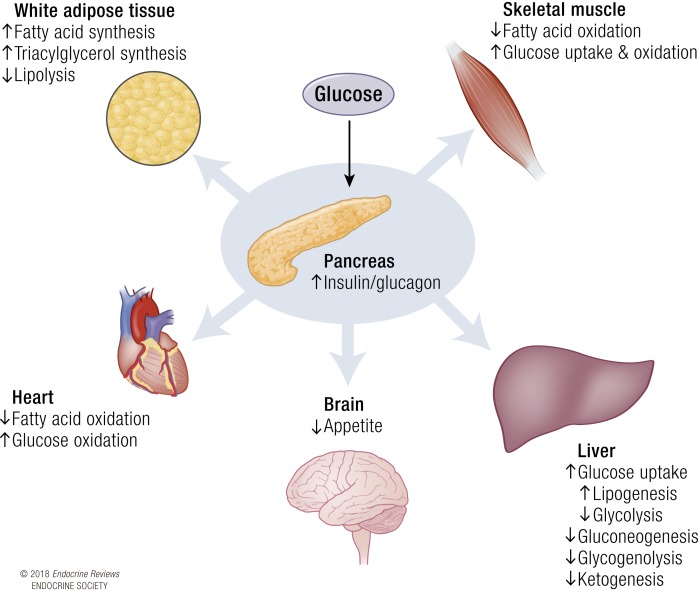
Systemic response to insulin. Upon glucose sensing, the pancreas increases the insulin/glucagon ratio. The rise in insulin stimulates many metabolic processes in the key metabolic organs: the liver, heart, brain, white adipose tissue, and skeletal muscle. Collectively, these metabolic processes switch metabolism from a preference of fatty acid oxidation to glucose uptake and oxidation.

On a systemic level, maintaining metabolic homeostasis during feeding or fasting relies on multiorgan coordinated control of available fuel. During the postprandial period of a carbohydrate-rich meal for instance, pancreatic *β* cells respond to the rise in nutrients by releasing insulin into the bloodstream, increasing the insulin-glucagon ratio (Fig. 1). Under the influence of insulin, the liver is triggered to absorb glucose from the circulation and stop glycogenolysis and gluconeogenesis. Skeletal muscle assists in glucose clearance as insulin receptor binding of insulin results in translocation of glucose transporters (GLUT), mainly GLUT4, to the plasma membrane, allowing glucose to enter the cell. Adipose tissue responds to insulin by decreasing the rate of lipolysis and stimulating fatty acid and triacylglycerol synthesis from lipids and glucose (18). Collectively, this buffering capacity ensures that the exposure of tissues to hyperglycemia is minimalized and that nutrients are stored in adipocytes for release and oxidation in times of scarcity. Fatty acids derived from a meal trigger adipose tissue to reduce nonesterified fatty acid release and prompt hepatocytes to reduce endogenous triacylglycerol release, together stimulating the clearance of circulating triacylglycerol (19).

During fasting, the decrease in circulating dietary carbohydrates and lipids and decline in insulin-glucagon ratio induces a switch toward FAO (20). Glucagon stimulates hepatic glycogenolysis and ketogenesis, and the decrease in insulin suppresses hepatocyte malonyl-coenzyme A (CoA) synthesis and lipogenesis, with concomitant activation of FAO (21, 22). After an overnight fast, ketone bodies are in the micromolar range (∼30 μM for women and ∼60 μM for men), which is not detected by most assays (23). This is important because it emphasizes that an overnight fast already induces changes in metabolic flexibility in healthy humans.

Hydrolytic processes in white adipose tissue (WAT) are increased converting stored triacylglycerol to free fatty acids that are used in the periphery as fuel, particularly in the skeletal muscle and heart. Additionally, lipolytic processes in the muscle aid in increasing lipid supply (16). Because adipose tissue is the predominant source of free fatty acids, the capacity of adipose tissue to store fatty acids during caloric availability and release fatty acids during caloric restriction is an important determinant of metabolic flexibility (24).

### Biochemical transition between feeding and fasting

**Figure 2.**
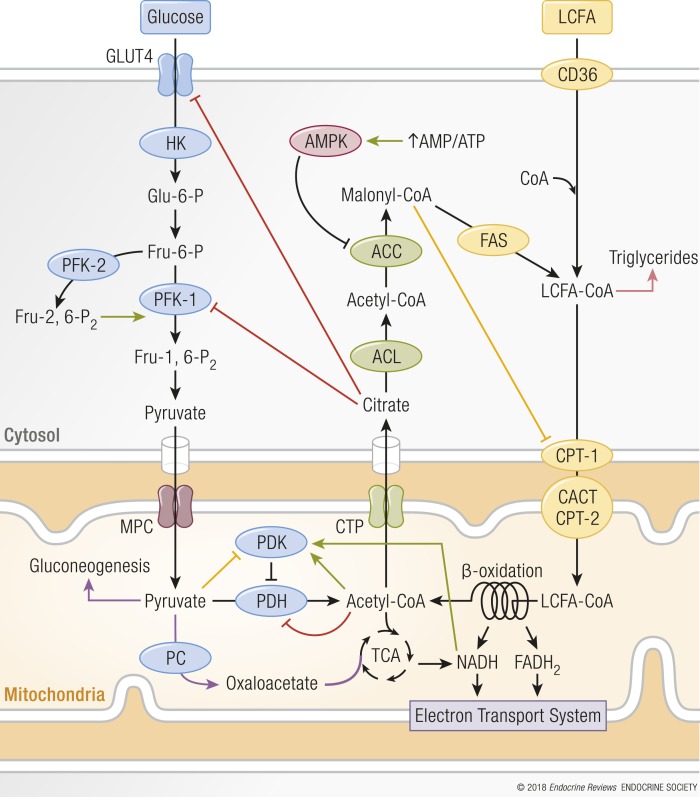
Mechanism of reciprocal inhibition of glucose and fatty acid oxidation. When glucose uptake and consumption increases, fatty acid oxidation is suppressed by malonyl-CoA’s allosteric inhibition of CPT-1, and increased pyruvate from glycolysis inhibits PDK, which stimulates glucose oxidation (yellow lines). CPT-1 inhibition increases the concentration of LCFA-CoAs, which then are used for triglyceride synthesis and stored (pink arrow). *Vice versa*, when fatty acid oxidation is high, glucose uptake, glycolysis, and pyruvate oxidation are decreased (red lines) because rising levels of acetyl-CoA and NADH impede PDH activity. Additionally, increased citrate levels inhibit GLUT4 and PFK-1. PFK-1 inhibition results in increased glucose-6-phosphate concentrations that inhibit HK. A decrease in pyruvate oxidation enables pyruvate to be used as either a gluconeogenic precursor or, in energetically demanding tissues, a substrate for PC, which produces oxaloacetate that is used as anaplerotic substrate (purple arrows). During caloric restriction, the rise in AMP/ATP activates AMPK, which inhibits ACC, stimulating fatty acid uptake by the mitochondria via CPT-1. ACL, ATP-citrate lyase; CACT, carnitine acylcarnitine translocase; CTP, citrate transport protein; CYTO, cytosol; FAS, fatty acid synthase; LCFA, long-chain fatty acid; MITO, mitochondria; MPC, mitochondrial pyruvate carrier; PC, pyruvate carboxylase. Green arrows indicate stimulatory reactions.

Cellular fuel selection depends on the type and amount of nutrient available. Cellular responses to the changes in nutritional state are predominantly assigned to the mitochondria (see “Mitochondrial function is essential for metabolic flexibility” and “Endocrine regulation of metabolic flexibility”). As proposed in Randle’s glucose-fatty acid cycle hypothesis, *in vitro* studies have shown that FAO is suppressed when glucose consumption is increased and *vice versa* (25) (Fig. 2). In the postprandial period of a carbohydrate-rich meal when glucose and insulin are high, glucose uptake, glycolysis, and pyruvate oxidation are favored and FAO is suppressed. These glucose-dependent processes increase the concentration of malonyl-CoA, which allosterically inhibits carnitine palmitoyltransferase-1 (CPT-1), which transports fatty acids into the mitochondria for *β*-oxidation. Fatty acids are then alternatively used for triglyceride synthesis and stored. Additionally, the rise in pyruvate from glycolysis inhibits pyruvate dehydrogenase kinase (PDK), which reduces the phosphorylation of pyruvate dehydrogenases (PDHs), resulting in an increased glucose oxidation (4).

On the other hand, during fasting, the inhibition of FAO is released by the action of the energy stress sensor adenosine monophosphate-activated protein kinase (AMPK), which inhibits acetyl-CoA carboxylase (ACC) by phosphorylation. Inhibition of ACC lowers malonyl-CoA concentrations, resulting in an increased activity of CPT-1 and amplified transport of fatty acids into the mitochondria for *β*-oxidation. When FAO is preferred, acetyl-CoA and reduced nicotinamide adenine dinucleotide (NADH) levels rise, impeding PDH catalytic activity through allosteric inhibition and via activation of PDK. During fasting, PDK gene expression increases through fatty acid–dependent peroxisome proliferator–activated receptor (PPAR) signaling (26). Additionally, an increase in fat availability elevates citrate levels, which inhibit phosphofructokinase and GLUT, impeding glucose uptake and use. Inhibition of phosphofructokinase also increases the cytosolic concentration of glucose-6-phosphate that inhibits hexokinase, making it more difficult to metabolize glucose (27). This allosteric inhibition results in a feed-forward loop, favoring FAO during times of nutrient scarcity to conserve glucose for biosynthetic processes and brain metabolism. Because glucose-derived pyruvate in the liver is no longer converted to acetyl-CoA by PDH, pyruvate can be used as a gluconeogenic precursor to avoid hypoglycemia.

In summary, the transition between feeding and fasting, and *vice versa*, is regulated by communication via endocrine cues and metabolic signals that orchestrates fuel oxidation and trafficking in tissues throughout the body. The allosteric inhibition of key regulatory enzymes in conflicting metabolic pathways ensures a robust and typically acute switch between the oxidation of glucose (fed state) or fatty acids (fasted state) (Fig. 2). On top of this direct regulation, a slower orchestration exists in which metabolic changes drive the activity of transcription factors and thereby fine tune the cellular metabolic responses.

### Prolonged fasting and caloric/dietary restriction

The importance of energy and nutrient sensing transcription factor regulated pathways can be demonstrated during prolonged fasting. It is somewhat surprising that, until 1967, ketone bodies (KBs) were thought to have no beneficial physiological role during prolonged fasting. Currently, we know that ketone bodies are produced by the liver upon prolonged fasting. The central nervous system requires approximately 140 g of glucose per day (equivalent to almost 600 kcal), also during fasting. Plasma KB then are an important source of energy because their blood levels and oxidation rates increase (28). During fasting, KB can act as an excellent respiratory fuel: 100 g of d-3-hydroxybutyrate yields 10.5 kg of ATP (22 ATP per molecule d-3-hydroxybutyrate), whereas 100 g of glucose yields only 8.7 kg of ATP (29). Ketogenesis occurs in the liver, where fatty acids undergo *β*-oxidation to form acetyl-CoA. This enters the ketogenesis pathway, where mitochondrial 3-hydroxy-3-methylglutaryl-CoA synthase is the most important enzyme involved in ketogenesis. This enzyme is inhibited by insulin; hence, ketogenesis only occurs at low insulin (and blood glucose) levels (30). Additionally, insulin inhibits lipolysis by inhibiting hormone-sensitive lipase in adipose tissue and thereby prevents the liberation of fatty acids for hepatic ketogenesis. More recently, ketone bodies have emerged as an alternative substrate to improve exercise performance (31), and could be used as a therapeutic target for patients with inborn errors of metabolism (32).

An alternative, and quantitatively less important, source of fuel during prolonged fasting is the breakdown of amino acids, in particular branched-chain amino acids (BCAAs). The mitochondrial branched-chain *α*-ketoacid dehydrogenase (BCKD) complex is the rate-limiting step in BCAA catabolism (33). BCKD can be allosterically inhibited when acyl-CoA concentrations and NADH levels are sufficient (34). This ensures that under fed conditions and during short intervals of fasting or light exercise, cellular proteins are conserved. With nutrient abundance, however, when BCAA are present in excess such as after a protein-rich meal, BCKD also becomes active as BCAA-derived *α*-ketoacids allosterically inhibit BCKDs deactivating kinase (35).

Dietary restriction, more commonly known as caloric restriction (CR), is the prolonged and controlled reduced intake of all dietary constituents while maintaining appropriate intake of vitamins and minerals. Recycling of cell-intrinsic macromolecules is essential to sustain metabolic processes when nutrients remain chronically scarce. This intricate salvaging process is controlled by autophagy (36). Autophagy is regulated by the deactivation of the nutrient sensors mechanistic target of rapamycin (mTOR) and v-Akt murine thymoma viral oncogene homolog 1/protein kinase B (Akt/PKB), and activation of the cellular energy status sensors AMPK and sirtuins (SIRTs) (37).

**Figure 3.**
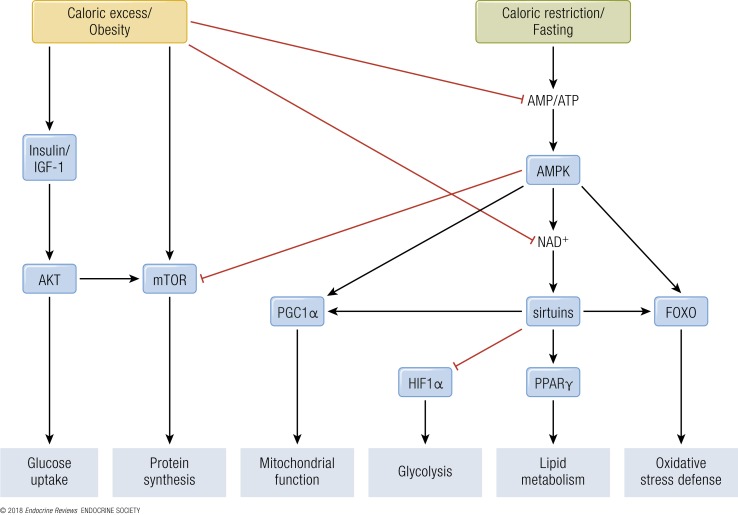
Energy- and nutrient-sensing transcription factor–regulated pathways. During caloric excess and obesity, anabolic processes are stimulated via the insulin/IGF-1 and TOR pathways. Additionally, caloric excess suppresses catabolic processes via a decrease in the AMP/ATP ratio, leading to reduced activation of AMPK and downstream activity of PGC1*α*, sirtuins, and FOXO. Simultaneously, decreased NAD^+^ levels reduce sirtuin activity, inhibiting PPAR*γ* activity and increasing HIF1*α* activity. Alternatively, during caloric restriction and fasting, catabolic processes are stimulated by the increase in AMP/ATP ratio and consequential activation of AMPK. AMPK also reduces anabolic processes through TOR inhibition. AKT, v-Akt murine thymoma viral oncogene homolog.

In mammals, reduced insulin/IGF-1 signaling through reduced nutrient intake inhibits Akt, which leads to activation of the forkhead box protein O (FOXO) transcription factors that upregulate the cells’ maintenance pathways: DNA repair, autophagy, and stress resistance (38). mTOR signaling stimulates growth and blocks tissue maintenance when nutrients are plentiful. However, upon CR, reduced intake of proteins, particularly of BCAA, downregulates the mTOR pathway, causing a switch toward salvage pathways such as autophagy and conserved translation (39). In response to increasing cellular AMP/ATP ratios, which rise during CR, AMPK activates catabolic pathways and represses anabolic pathways (40). In parallel, SIRT activity is increased during CR, which depends on oxidized nicotinamide adenine dinucleotide (NAD^+^) concentrations, and leads to protein deacetylation and improved mitochondrial function (see “*SIRTs*”; Fig. 3). Although much work has been performed on CR and the metabolic adaptations thereof, it is currently unknown if this would be a suitable strategy in human interventions.

### Caloric excess

With caloric excess, the mitochondria are overwhelmed by an excess in in substrates derived from fatty acids, glucose, and amino acids. In this setting, metabolic flexibility is altered by a push-mechanism (see “Defining Metabolic Flexibility”). Through persistent allosteric inhibition and feed-forward responses this surplus leads to mitochondrial metabolic “indecision” and ineffective substrate switching, resulting in incomplete substrate utilization for energy production and subsequent storage of substrates in (ectopic) depots (4). For instance, high levels of fatty acids can increase expression of PDK through transcriptional activation of PPAR*α*, resulting in inactivation of PDH and thus blunting glucose oxidation (41). It has been suggested that a low ATP utilization in combination with a high mitochondrial membrane potential increases reactive oxygen species (ROS) production, causing oxidative damage and triggering signaling events, and importantly affects the activity of redox sensitive metabolic enzymes (4).

With a low electron flux through the electron transport system, the NADH/NAD^+^ ratio in the mitochondria increases causing redox inhibition of several tricarboxylic acid (TCA) cycle enzymes and reduces the supply of carbons into the TCA cycle. This results in elevated levels of acetyl-CoA and other acyl-CoA at crucial sites where catabolism of fatty acids, glucose, and amino acids converge, leading to a state in which upstream substrate catabolic flux slows down considerably, a phenomenon coined as mitochondrial metabolic “gridlock” (4). Furthermore, in times of caloric excess, elevated levels of acetyl-CoA increase protein acetylation events (because they serve as acyl donors). A high NADH/NAD^+^ ratio leads to inactivation of mitochondrial and nuclear SIRT deacetylases, reducing mitochondrial biogenesis and activity (42). Indeed, prolonged high-fat feeding of rodents or continuous fatty acid exposure of myotubes leads to a reduction in nuclear-encoded mitochondrial genes (43). A reduction in *β*-oxidation can cause accumulation of long-chain fatty acyl-CoA, diacylglycerol, triacylglycerol, and ceramide, which may increase serine phosphorylation of the insulin receptor and reduce activation of Akt/PKB, leading to impaired insulin signaling (44). Ultimately, metabolic flexibility is impaired during long-term caloric excess, affecting many tissues. Among other clinical phenotypes, caloric excess is correlated to metabolic syndrome, obesity, T2DM, and cardiovascular disease (4) (see “Pathophysiology of Metabolic Inflexibility”; Fig. 3).

### Physical exercise

A good example of cell intrinsic metabolic programming upon physiological stimulation occurs in skeletal muscle. Skeletal muscle consists of oxidative (type I) and glycolytic (type II) fibers, which differ in their metabolic abilities. Oxidative muscle fibers have a high mitochondrial density; hence, they prefer oxidative phosphorylation for ATP production. They also contain more lipid droplets and rely on FAO. Glycolytic muscle fibers have a low mitochondrial density and rely predominantly on the breakdown of stored glycogen by glycolysis for their ATP production (45, 46). During low-intensity exercise, oxidative muscle fibers predominantly rely on FAO for their ATP production. During more intense exercise, the rising ATP utilization rate induces a metabolic switch from FAO to glucose metabolism. When maximal pyruvate production outstrips its mitochondrial import during severe intensity exercise, pyruvate is converted into lactate and NAD^+^ by lactate dehydrogenase in the cytosol, after which lactate is excreted from the cell. NAD^+^ generation helps maintain cytosolic redox potential and promotes substrate flux through glycolysis to sustain ATP generation (47).

As such, regular physical exercise is a classic example of how metabolic flexibility is regulated by transcription factors. During acute exercise, an increased AMP/ATP ratio, sensed by AMPK, increases transcription, translation, and activity of the transcriptional coactivator PPAR gamma coactivator 1-alpha (PGC1*α*) (40, 48). PGC1*α* is a regulator of exercise-induced adaptations in the capacity of oxidative phosphorylation (OXPHOS) in skeletal muscle (49, 50). In particular, PGC1*α* interacts and coactivates many transcription factors and nuclear receptors that are involved in mitochondrial energy homeostasis and metabolic adaptations, such as nuclear respiratory factors (NRFs) and PPARs. NRFs regulate the expression of nuclear genes encoding OXPHOS proteins, and PPARs regulate the transcription of genes that encode enzymes involved in lipid transport and catabolism (51). The increase of mitochondrial biogenesis and FAO improves insulin sensitivity. The role of PGC1*α* in metabolic flexibility is underlined by observations that basal PGC1*α* skeletal muscle expression is reduced in sedentary subjects (49).

In mouse muscle cells, exercise-dependent calcium influx activates calcineurin that dephosphorylates the helix-loop-helix leucine zipper transcription factor EB (TFEB) ensuring its localization to the nucleus. Here, TFEB controls the expression of genes involved in glucose uptake such as GLUT1 and 4, hexokinase (HK) I and II, TBC1 domain family member 1, and glycogen synthase, which collectively lead to glycogen production to sustain energy generation during later bouts of exercise (52). Additionally, TFEB increases the expression of NRF2 and mitochondrial transcription factor A (TFAM), which are regulators of mitochondrial biogenesis in muscle. Specifically, TFAM regulates mitochondrial DNA (mtDNA) transcription and replication (52). Recently, TFEB was found to act independently of PGC1*α* to promote mitochondrial biogenesis and oxidation of glucose and lipids, and because TFEB directly alters cellular glucose handling via GLUT1/4 expression and insulin sensitivity via nitric oxide (NO) synthase, it is considered a critical player of metabolic flexibility during physical activity (52).

### Hibernation and cold exposure

Research in migratory birds (53), killifish (54), and in animals that enter states of torpor or hibernation (55) has revealed that temperature can influence metabolic reprogramming and metabolic flexibility. Cold-acclimated birds increase thermogenic capacity through elevated expression of fatty acid transporter proteins in flight-muscle fibers and mitochondrial membranes, and increase mitochondrial density and FAO, OXPHOS, and TCA cycle enzymes (53). Metabolism in hibernators is also switched to lipids as the major fuel for all organs, although their overall metabolic rate is severely reduced (55). Moreover, catabolic processes that consume large amounts of ATP are suppressed, including mitosis and cell proliferation, mitochondrial metabolism, transmembrane ion transport, global mRNA transcription, and protein biosynthesis. Protein biosynthesis in particular is linked to decreased Akt and mTOR activity (55). These studies cautiously suggest that reducing the core body temperature in mammals may have a beneficial effect on metabolic health.

Until recently the presence of brown adipose tissue (BAT) was thought to be confined to small mammals and infants. However, recent studies have shown that in adult humans, BAT activity can be stimulated by mild cold (16°C) exposure, suggesting that BAT has physiological relevance in humans, too (56–58). Importantly, brown adipogenesis has been reported in WAT, demonstrating that WAT harbors the potential to switch to BAT. Brown-like adipocytes within WAT are known as brite (brown-in-white) or beige adipocytes, and transdifferentiation from white to brown is commonly referred to as “browning.”

Master regulators of browning in humans are PPAR*γ* and PGC1*α* (59). PPAR agonists in white adipocytes caused conversion to a brite/beige molecular phenotype *in vitro*, defined by increased mitochondrial oxygen consumption and amplified expression of the thermogenic gene program, including uncoupling protein 1, which is located in the inner mitochondrial membrane and uncouples mitochondrial respiration, releasing it as heat (60). In mice, transcriptional changes in response to thermogenic challenges suggest increased glucose uptake, glycolysis, glycogen metabolism, pentose phosphate pathway (PPP) flux, and OXPHOS (61), although the exact contribution of such pathways to thermogenesis is unclear. In bright/beige adipocytes glucose, utilization is likely switched from OXPHOS to glyceroneogenesis favoring triglyceride synthesis. The shift from glucose to fatty acid use for OXPHOS is enforced by increased *β*-oxidation of fatty acids, which restricts complete oxidation of glucose through PDK inhibition of the PDH complex. This metabolic pathway rewiring directs pyruvate toward glyceroneogenesis (60).

Obese subjects showed lower BAT activity than lean subjects, and low BAT activity is associated with metabolic dysfunctions such as T2DM and aging (62). Although noradrenalin can induce browning, adrenergic therapy does not activate BAT to the same extent as cold exposure, and incurs adverse cardiovascular effects (59). It seems that increasing BAT activity and induction of browning by short-term cold exposure in humans can increase insulin sensitivity (63), but more work needs to be performed before it can be regarded as a suitable treatment of obesity and T2DM.

### Interplay between circadian rhythm and metabolic flexibility

The circadian clock enables organisms to anticipate the diurnal variation in metabolic substrates (64). The circadian clock can be divided into the central and peripheral clocks. The central, or master clock, is located in the hypothalamic suprachiasmatic nucleus and can function autonomously without any external input, but can be entrained by temporally relevant external input such as, temperature, light, and feeding/fasting cycles (65). The central circadian pacemaker mainly acts through its powerful influence over the endocrine system (66). The peripheral clocks are synchronized by the central clock and are present in almost all mammalian tissues, where they regulate tissue specific gene expression (65).

Indeed, the circadian clock can have major effects on metabolic flexibility and is even able to coordinate temporal and spatial organization of lipids and circadian rhythmicity of mitochondrial function (67, 68). The peripheral circadian clock has several mechanisms to influence metabolism including regulation of metabolite levels, interaction with nutrient sensors, control of rate-limiting metabolic enzymes, and modulation of nuclear receptors (65). Circadian proteins directly coordinate with key regulators of nutrient homeostasis, including AMPK, cAMP response element-binding protein, and Akt/PKB, which are themselves driven by daily rhythms of nutrient supply (69, 70). Gene expression of key metabolic enzymes show diurnal variations (71). Expression of nuclear receptors such as members of the PPAR family and estrogen-related receptor family are also under circadian clock control (65). Moreover, diurnal variations in human skeletal muscle oxidative capacity were recently observed and may be linked to the circadian clock in muscle (72).

The circadian clock is under influence from, and can synchronize to, external stimuli such as food intake and diet (64). For instance, the liver synchronizes its peripheral circadian rhythm based on the availability of circulating metabolites (71, 73). Peripheral tissues communicate dietary signals to the brain via endocrine cues such as ghrelin, leptin, and insulin, meaning feeding rhythm strongly contributes to the reciprocal relationship of the circadian clock and metabolism (74, 75). The nutrient sensors AMPK and SIRT1 that modulate circadian gene expression and circadian oscillating metabolites such as cAMP and NAD^+^, are known regulators of the circadian clock (64, 65, 76).

Although the expression of many genes of both the circadian clock and metabolism fluctuate reciprocally and in response to environmental cues, enzymatic activity can be modulated by posttranscriptional modification of proteins, which adds an additional layer of rhythmicity to the circadian network (75). Research in this field is very scarce and much is still unknown. Not only the timing but also the composition of food intake affect the circadian clock. Mice fed on high-fat diets, for instance, showed altered expression of core clock genes and the genes under their control, altered circadian rhythms, and consumed larger amounts of food during their active phase (77). The extent to which metabolites have control over the circadian clock is currently unknown. It is likely, however, that an interplay of all metabolites (the metabolome) acts as a cue for the circadian clock because many metabolites affect its phase, amplitude, and/or period of oscillations (75).

Disruption of the circadian rhythm, or circadian misalignment, in human subjects can result in insulin resistance (78). As a result, (night)shift workers are at a greater risk to develop obesity, T2DM, cardiovascular disease, and metabolic syndrome (79, 80). Currently, much more work is required to fully understand the mechanistic link between a disruption in the circadian rhythm, the loss of metabolic flexibility, and the development of metabolic disease.

### Aging

The underlying multifactorial aspects to aging make it difficult to discern specifically which are most causative of the aging process. Nonetheless, understanding the fundamental aspects of aging and targeting these processes using physiological or pharmacological approaches can limit the progression of many age-related diseases (39).

The metabolic influence on aging and lifespan has gained increased attention over the past decade; as such, major regulators of metabolic flexibility play dominant roles in aging (81, 82). Indeed, metabolic flexibility is negatively correlated with aging (83) and targeting metabolic flexibility as a cause for aging and related comorbidities may provide cues to delay the onset of age-related diseases and prolong health span. Mitochondrial dysfunction and a cellular shift toward a glycolytic phenotype is intimately linked to senescence and the “senescence-associated secretory phenotype” that entails the secretion of multiple factors such as proinflammatory cytokines, proteases, and growth factors that have potent local and systemic effects such as inflammation and metastasis (84, 85). Perturbation of mitochondrial function and nutrient-sensing pathways, particularly related to glucose homeostasis, is a hallmark of aging (86). As such, the nutrient and energy sensing pathways (insulin/IGF, mTOR, AMPK, and SIRTs) are causally involved in fitness and longevity (39, 87). Consistent with this, CR increases health span and/or lifespan in model organisms and improves several markers of health in humans (38) (see “Prolonged fasting and caloric/dietary restriction”).

It is currently unclear how metabolic flexibility is perturbed in the elderly, because few metabolic flexibility studies have been conducted in late middle-aged and aged populations. One underlying cause to age-related metabolic pathophysiology is a sedentary lifestyle that is steadily increasing in prevalence in the general population and in particular strongly increases as people age. A study in middle-aged postmenopausal women showed that endurance training improved work-related ability to mobilize and oxidize free fatty acids, suggesting that in the elderly metabolic flexibility can still be trained (88). Importantly, exercise is a principal preventive strategy to improve metabolic flexibility and prolong healthy aging (89) (see “Exercise training”).

In summary, on a cellular level, acute metabolic flexibility is a universal property of healthy cells (1). Metabolic flexibility, and therefore substrate flux, is principally determined by the reciprocity of metabolic circuitries, of which the presence is dependent on the cell’s gene and protein expression, nutrient availability and/or demand. On a systemic level, metabolically active organs such as the liver, muscle, heart, and adipose tissue, communicate to best organize the utilization of available fuel. This holistic and *vis-à-vis* orchestration of available nutrients to sustain whole-body energy homeostasis has ensured organism survival and is therefore interwoven with both healthy and diseased states of metabolism.

## Regulation of Metabolic Flexibility

The intrinsic qualitative and quantitative capacity of cells to oxidize or store energy is dependent on the molecular organization of their metabolic pathways. This tissue-specific metabolic programming depends on the coordinated action of various enzymes and transcription factors, which are collectively orchestrated by intrinsic mitochondrial function ), circulating endocrine factors, and epigenetic programming.

### Mitochondrial function is essential for metabolic flexibility

Mitochondria are equipped to transform metabolism intermediates such as pyruvate, fatty acids, and amino acids, into the reduced energetic equivalents NADH and/or flavin adenine dinucleotide through *β*-oxidation and the TCA cycle. These are then used in complexes located in the inner mitochondrial membrane where electrons are transferred from electron donors to acceptors by redox reactions, with oxygen as their ultimate acceptor. During this transfer to a lower redox potential, the liberated energy is used to extrude protons from the mitochondrial matrix into the mitochondrial intermembrane space, generating an electrochemical proton gradient that is used by the F_1_F_o_-ATP-synthase to generate ATP from ADP and inorganic phosphate. As such, mitochondria are the final acceptors for metabolic substrates and are the main players for understanding the pull concept of metabolic flexibility.

Mitochondria are pliable organelles, and they adapt their morphology to nutrient availability and in doing so regulate OXPHOS activity and substrate preference (90, 91). Recently, isolated skeletal muscle mitochondria from rats fed high-sugar or high-fat diets showed reduced metabolic flexibility, indicating that substrate preference is independent of cytosolic-mitochondrial communication and in fact a consequence of inherent mitochondrial biochemical network interactions (92, 93).

Mitochondrial bioenergetic function can be controlled through both acute changes, aimed to promptly modify activity, and longer term transcriptional responses, aimed to regulate mitochondrial volume density. Mitochondrial bioenergetics can be altered through calcium activation of mitochondrial enzymes (94), posttranslational mechanisms such as protein acetylation, and through dynamic adaptations of morphological architecture by use of mitochondrial fission and fusion components (95). For instance, the acetylation state of mitochondrial proteins differs strongly between the fed and fasting state (96).

Mitochondrial morphology is dependent on nutritional status of the cell. Cells exposed to a nutrient overload have a fragmented mitochondrial network, whereas upon CR, mitochondria appear more interconnected (95). Increasing mitochondrial elongation and interconnectivity induces a bioenergetic adaptation that increases ATP synthesis capacity and efficiency (97). Conversely, fragmentation of the mitochondrial network reduces bioenergetic efficiency and might protect against detrimental effects of nutrient overload (98, 99).

Many studies support the idea that deregulation of mitochondrial function underlies the onset of metabolic inflexibility [reviewed in Muoio (4)], although a causal link between the two still remains to be fully established (100, 101). Besides regulating glucose metabolism and fatty acid oxidation in most cell types, mitochondria regulate triglyceride synthesis and gluconeogenesis in hepatocytes and lipolysis in adipose tissue (102, 103). Additionally, insulin secretion from pancreatic *β* cells (104) and synthesis and secretion of adipokines from WAT is dependent on mitochondrial function (105), suggesting that mitochondria fulfill a crucial role in determining cellular, tissue, and systemic metabolic flexibility. Furthermore, a reduction in mitochondrial oxidative capacity is linked to the development of T2DM and obesity (see “Metabolic syndrome and T2DM”), whereas exercise-enhanced mitochondrial performance is related to a better metabolic flexibility and insulin sensitivity (106) (see “Cancer”).

### Endocrine regulation of metabolic flexibility

The importance of endocrine regulation of metabolic flexibility, in particular to coordinate complex interorgan government of energy storage and oxidation, is undeniable. As discussed previously, metabolic flexibility relies on the action of insulin and glucagon major regulators of glucose metabolism in response to dietary stage (107) (see “Feeding/fasting”, “Biochemical transition between feeding and fasting”, and “Prolonged fasting and caloric/dietary restriction”). Other newly discovered paracrine and endocrine factors have emerged that also alter metabolism, and some of the most important will be discussed here.

#### Gut endocrine regulation of metabolic flexibility

The postprandial state is characterized by various, mainly gut-derived, factors that somehow affect metabolism. The postprandial increase of some of these actually modulates metabolic flexibility either directly or indirectly via increased insulin secretion. For example, glucagonlike peptide-1 (GLP-1) is released by enteral L cells and agonizes pancreatic insulin secretion. However, GLP-1 exerts direct inhibitory effects on hepatic glucose production via direct hepatic or neuronal inhibition (108). Also, GLP1 may contribute to reduced intestinal lipoprotein production. Likewise, bile acids also facilitate nutrient trafficking in a hormone-like fashion (109). Bile acids induce insulin secretion directly via the transmembrane bile acid receptor Takeda G protein–coupled receptor 5 (on *β* cells and indirectly via stimulation of l-cell derived GLP-1 and subsequent insulin release (110–112). Additionally, the postprandial increase in bile acids also increases insulin sensitivity and energy expenditure (109). In contrast to these two examples, the peptide ghrelin is produced in stomach X/A cells and actually decreases during food intake. The postprandial decrease in ghrelin lowers hepatic glucose production while increasing peripheral glucose-uptake in both skeletal muscle and adipose tissue (113). All in all, the combined effects of different factors during food-intake and fasting facilitate the organisms’ fuel availability and metabolic flexibility.

“Myokines play important roles in mediating the positive effects of exercise on whole-body metabolism.”

#### Other endocrine factors affecting metabolic flexibility

**Figure 4.**
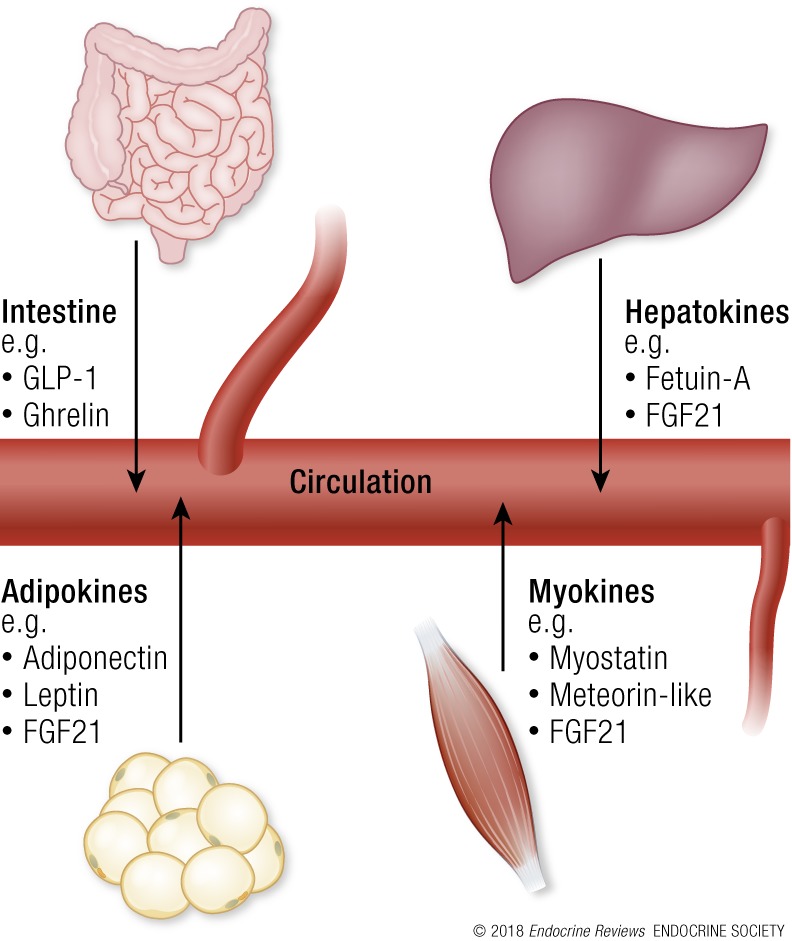
Circulating factors not produced by specific endocrine glands are involved in metabolic flexibility. Examples include those that are produced by the intestine, adipocytes (adipokines), muscle (myokines), and liver (hepatokines) and released into the bloodstream. These endocrine factors act on metabolism through paracrine and endocrine signaling, and distal organs include skeletal muscle, adipose tissue, liver, pancreas, heart, and brain. Much is currently unknown about these endocrine factors. See “Other endocrine factors affecting metabolic flexibility” for a brief description of some examples and their role in regulation of metabolic flexibility.

Many other circulating factors are involved in metabolic flexibility, including cytokines and other peptides that are expressed, produced, and released by adipocytes (adipokines), muscle (myokines), and liver (hepatokines). Although the precise function of many of these factors remains elusive, some exert autocrine, paracrine, or endocrine effects that are fundamental for organ cross-talk in the regulation of energy homeostasis (114). For a comprehensive view on the roles of adipokines, myokines, and hepatokines, we refer the reader to some excellent reviews on the topic (114–116). In this section, we give brief examples to highlight their role in regulation of metabolic flexibility. Figure 4 summarizes these examples.

Fully functional adipocytes reduce lipotoxicity in tissues such as the heart and liver and they maintain a healthy balance of adipokines, which exert paracrine effects on adipocytes in their direct vicinity and endocrine effects on the central nervous system, immune system, and peripheral tissues. Adiponectin suppresses glucose production in the liver and enhances FAO in skeletal muscle (117, 118). Indeed, high levels of the insulin-sensitizing, antiapoptotic, and anti-inflammatory hormone adiponectin has been proposed to improve metabolic flexibility of adipose tissue, enhancing its function under metabolic challenges (119). Leptin is a central feedback indicator for the brain on the amount of stored energy in the body and rises in concert with the amount of adipose tissue (120). Leptin also exhibits diurnal expression patterns that are dampened in obese subjects (see “Interplay between circadian rhythm and metabolic flexibility”) (121). Taken together, adipocytes have an important role in systemic neuroendocrine regulation of metabolic flexibility as adipose tissue is both responsive to and responsible for diverse metabolic, inflammatory, and hormonal signals [reviewed in Luo and Liu (115)].

Myokines provide skeletal muscle with the ability to mediate whole-body metabolism via endocrine signaling to adipose tissue, liver, pancreas, heart, and brain (122). In particular, myokines play important roles in mediating the positive effects of exercise on whole-body metabolism [reviewed in Oh *et al.* (114)]. Production of myokines is predominantly influenced by skeletal muscle contraction and can alter glucose disposal, FAO, and lipolysis. For instance, the alteration of myostatin expression contributes to the proliferation, development, and metabolism of adipose and skeletal muscle tissue (122). Exercise decreases the expression of myostatin in humans and obesity is associated with increased myostatin expression (122). Moreover, myostatin knockout mice have significantly improved insulin sensitivity and glucose uptake, have increased peripheral tissue FAO, and are protected from diet-induced obesity (123, 124). Another circulating factor named meteorin-like has been described to be released from skeletal muscle after exercise and in adipose tissue upon cold exposure (125). Meteorin-like is involved in the adaptive responses to the regulation of energy homeostasis and tissue inflammation, but the therapeutic potential for metabolic and inflammatory diseases is currently unknown. Irisin, another myokine, has been proposed as an important glucoregulatory candidate. However, contradictory findings concerning the role of irisin in humans exist; therefore, results must be interpreted with caution.

Hepatokines can also regulate whole-body metabolic flexibility and some are even considered as potential targets for the treatment of cardiovascular disease [reviewed in Jung *et al.* (116)]. For example, fetuin-A has a major role in the regulation of insulin sensitivity as fetuin-A–deficient mice showed improved insulin sensitivity (116). Nucleotide polymorphisms in human fetuin-A and high levels in serum are a predictable marker for the incidence of T2DM (116). Additionally, fasting reduces the circulating levels of fetuin-A, whereas high levels of saturated fatty acids and glucose augments the expression of fetuin-A (116). Mice deficient of fetuin-A are resistant to high-fat diet–induced obesity and have improved glucose tolerance (114).

Interestingly, some of these paracrine and endocrine factors are secreted by multiple tissues, and their local function and impact on metabolic flexibility can depend on their origin and local plasma concentrations. IL-6, for instance, when secreted by skeletal muscle, stimulates AMPK activity and in this way increases glucose uptake and *β*-oxidation in muscle and adipose tissue (114). IL-6, is, however, also secreted by adipocytes from obese patients and negatively affects metabolic flexibility by decreasing insulin signaling and glucose uptake because of its proinflammatory properties (114, 123). Another example is fibroblast growth factor 21 (FGF21), which, as a myokine, increases GLUT1 expression in skeletal muscle, boosting glucose uptake (114). FGF21 from muscle also exerts endocrine-like effects on WAT, increasing lipolysis and *β*-oxidation and inducing browning (126). As an adipokine, however, FGF21 stimulates insulin-independent glucose uptake in peripheral tissues; as a hepatokine, FGF21 stimulates lipolysis in WAT (114).

Although the role of adipokines, myokines, and hepatokines in the control of whole-body energy homeostasis and metabolic flexibility is only recently becoming evident, it is to be reported that they have clinical relevance and diagnostic potential.

### Epigenetic regulation of metabolic flexibility

Metabolic flexibility in response to environmental stimuli, such as diet and exercise, are dramatically influenced by epigenetic factors as they influence gene expression by regulating access of transcriptional machinery to DNA. Evidence that epigenetic changes drive metabolic inflexibility in humans is emerging (127).

Metabolic networks, in particular those in the mitochondria, directly transmit information about the cells metabolic state to epigenetic programming enzymes that, for instance, add or remove epigenetic markers onto chromatin (128, 129). In this way, histones can act as metabolic sensors, converting changes in metabolism into stable patterns of gene expression, a concept named “metabolic memory” (130, 131). Both global fluctuations in metabolite levels caused by nutritional inputs, circadian rhythm, and oxygenation, or local changes depending on intracellular metabolite distribution, can translate into epigenetic changes (130, 132). The abundance of cofactors and the metabolic enzymes that generate them not only alter epigenetic enzyme histone modification, but also affect DNA methylation and posttranslational modification of the epigenetic enzymes themselves, resulting in a complex feedback network (42, 133). Moreover, the amplitude and duration of the metabolic stimulus required to alter the epigenome is dependent on the vastly different kinetics of epigenetic modifier enzymes (130). Metabolic sensory epigenetic programming enzymes include: histone acetyltransferases that acetylate histones using acetyl-CoA (133), SIRTs that deacetylase histones using NAD^+^ as a cofactor (42), histone methyl-transferases that methylate or remove methyl marks using *S*-adenosylmethionine as a methyl donor, or lysine-specific demethylases that use flavin adenine dinucleotide as a cofactor (134). How these epigenetic regulators are targeted to specific sites, such as promotor regions, how transient their epigenetic markers are, and how these changes are inherited, is still under active investigation (135).

People with a family history for T2DM have an increased risk for developing metabolic inflexibility; the lower HK II activity and PGC1 expression play a role in this (136). For instance, skeletal muscle from families with a history of T2DM has altered methylation status of genes involved in muscle function and insulin and calcium signaling (137). Tissue-specific epigenetic regulation may be of particular importance for metabolic flexibility because overweight patients with T2DM also have hypermethylated promoter regions of PGC1*α* and an OXPHOS complex I subunit in skeletal muscle (137). Promoters of many genes that are important for pancreatic *β*-cell survival and function are differentially methylated in T2DM patients compared with controls (137). Moreover, obese patients have an altered epigenetic landscape associated with disrupted lipid oxidative metabolism and mitochondrial function in adipose tissue, skeletal muscle, and liver (138).

## Pathophysiology of Metabolic Inflexibility

Although inborn errors of metabolism are clear examples of metabolic inflexibility, here we focus on acquired metabolic inflexibility. For specific information on inborn errors of metabolism we refer the reader to a comprehensive book on the subject (139). Here, we discuss the pathophysiology of metabolic flexibility in the context of obesity, metabolic syndrome and T2DM, as well as systemic inflammation, cardiovascular disease, and cancer.

### Obesity

An estimated 45% of the adult US population is obese or overweight (140). At the heart of obesity lies the inability to regulate lipolytic and antilipolytic processes in adipose tissue during starvation and feeding, respectively. Obesity is predominantly associated with elevated levels of plasma free fatty acids (141). High circulating levels of free fatty acids inhibit glycogen synthase activity and PDH activity, which leads to reduced disposal and oxidation of glucose. Besides adipocyte metabolic dysfunction, skeletal muscle mitochondrial capacity and *β*-oxidation are reduced. Specifically, upregulation of PPAR*α* and its downstream targets in response to high-fat feeding are defective (142). Excess calories are then stored in peripheral fat depots as triglyceride; when these depots reach their maximum capacity and fail to expand, fat accumulates in ectopic depots, including skeletal muscle and the liver. Ectopic fat deposition is related to metabolic abnormalities and defects in insulin sensitivity, T2DM, cardiovascular disease, and cancer (143). Finally, obesity is associated with a state of chronic low-grade inflammation because ectopic fat depots release more inflammatory mediators than peripheral fat depots and infiltration of macrophages (24). Metabolic inflexibility and fat deposition therefore likely reinforce one another in a vicious cycle.

### Metabolic syndrome and T2DM

Metabolic syndrome is defined by visceral obesity and at least two of the following factors: elevated blood pressure, raised fasting plasma glucose concentration, elevated triglyceride concentrations, and/or low high-density lipoprotein cholesterol. Together with excess body fat and physical inactivity, metabolic syndrome is a major risk factor for developing T2DM and related complications include cardiovascular disease; increased rates of specific cancers, physical, and cognitive disability (5); and is associated with increased risk for T2DM and cardiovascular disease, among cancer (144). Consequently, individuals with metabolic syndrome have increased mortality and a shortened lifespan (145).

The best example of compromised metabolic flexibility in metabolic syndrome is a deteriorated insulin-mediated substrate switching. As such, metabolic inflexibility is at the core of the pathophysiology of insulin resistance (146). After a high-fat meal, patients with metabolic syndrome have higher levels of glycaemia and lower skeletal muscle free fatty acid uptake compared with healthy individuals. In response to fasting, skeletal muscle from patients with insulin resistance are less able to switch to FAO compared with healthy individuals (146). An increased dependency on glucose oxidation and decreased reliance on FAO in offspring from patients with T2DM suggests that impaired FAO may precede insulin resistance (146–148).

Moreover, studies strongly imply that impaired mitochondrial function precedes insulin resistance (41, 149). The importance of OXPHOS and its maintenance in relation to insulin resistance is underscored by observations that skeletal muscle mitochondria from patients with T2DM or obesity are unable to increase replication of mtDNA, which encodes essential OXPHOS components, in response to exercise combined with CR (150). Moreover, skeletal muscle mitochondria from insulin-resistant patients have lower expression of PGC1*α* and its downstream targets, and differ in mass, morphology, and function (150). In particular, muscle mitochondria from patients with T2DM show reduced expression of mitofusin-2, which regulates mitochondrial outer membrane fusion, and thus mitochondrial dynamics and quality control (151). They also have a lower maximal oxidative capacity, smaller mitochondria and reduced NADH oxidase (complex I) activity (152)

Interestingly, studies have demonstrated that BCAA and associated metabolites are strongly associated with insulin resistance and T2DM (153). Based on the theory of mitochondrial metabolic gridlock and anaplerosis, excessive BCAA metabolites are proposed to clog the *β*-oxidation machinery, particularly in skeletal muscle and liver, and thus contribute to accumulation of incompletely oxidized intermediates of fatty acids, particularly in the presence of a high-fat diet. Collectively, under these conditions, such byproducts render glucose superfluous as a substrate and, combined with the upsurge in ROS, can lead to insulin resistance (154).

### Systemic low-grade inflammation

One of the hallmarks of metabolic syndrome is low-grade chronic systemic inflammation (155, 156). In the case of obesity and insulin resistance, systemic inflammation can trigger and propagate metabolic inflexibility. Systemic inflammation and metabolic inflexibility can cause a vicious circle because metabolic inflexibility can also trigger systemic inflammation. How this is regulated at the cellular and molecular level is currently unknown, but hyperglycemia-induced mitochondrial ROS production can stimulate inflammation by signaling factors (157), such as protein kinase C, p38 MAPK, and c-Jun-*N*-terminal kinase (158).

Systemic low-grade inflammation as a trigger of metabolic inflexibility is best described in the context of obesity and lipid toxicity (159). As a result of excess fatty acid intake, organs that reach the maximum of their storage capacity and ectopic tissues that accumulate fatty acids upon overspill can become infiltrated by immune cells resulting in inflammatory processes. Dysregulated release and storage of fatty acids can lead to an increased release of inflammatory cytokines such as TNF*α* and monocyte chemoattractant protein-1 and decreased secretion of anti-inflammatory adipokines such as adiponectin. This can result in recruitment of M1 type macrophages and T cells. Additionally, B lymphocytes, neutrophils, eosinophils, mast cells, and natural killer cells have all been implicated in adipose tissue dysfunction. This lipid toxicity can therefore generate signaling intermediates that can interfere with local and systemic immune responses, causing a vicious cycle of immune-metabolic degradation (160).

Although the mechanism and specific mediators in lipid-induced inflammation are not completely understood, the endoplasmic reticulum (ER) is central to these responses because this is where both lipid biosynthesis and esterification processes as well as inflammatory pathways converge. Disrupted lipid synthesis in the ER can change ER membrane composition, leading to ER stress, dysfunction, and ultimately cell death, triggering inflammation (160). Lipids are also able to instigate inflammatory processes through interaction with cell-surface receptors, such as Toll-like receptor-4, and stress kinases in the cytoplasm, such as protein kinase R that through downstream signaling can induce the expression of genes that mediate inflammation and apoptosis, and promote inflammasome activity. Moreover, there is emerging evidence that lipids engage intracellular signaling pathways via protein kinase C isoforms that are related to T-cell activation and LPS responses (160). It is unlikely, however, that one of such responses underlies lipotoxicity, but that a combination of factors mediate lipid-associated inflammation (155, 156).

### Metabolic flexibility in immune responses

Metabolic flexibility and the accompanied rerouting of metabolic flux are essential for immune function. Following immune stimulation, naive lymphocytes that rely on *β*-oxidation of fatty acids and pyruvate oxidation via the TCA cycle become active and engage in glycolysis and glutaminolysis (161). During glycolysis, glucose is metabolized to lactate in the presence of oxygen, which allows for augmented rates of glycolytic flux and regeneration of NAD^+^. Additionally, the switch to glycolysis enables glycolysis and TCA cycle intermediates to be used as key sources of carbon molecules for biosynthesis of nucleotides, amino acids, and lipids. In this way, glycolysis facilitates robust growth, rapid cellular proliferation, and the production of large quantities of effector molecules, ultimately to mount a sufficient immune response. The exact molecular regulation and thus the dependency on this metabolic switch differs between specific lymphocyte subsets (161).

Reminiscent of the characteristic metabolic network adaptation of tumor cells (see “Cancer”) myelocytomatosis oncogene (Myc)–regulated glutamine dependency is important for activated lymphocytes because glutamine fulfills a vital role as anaplerotic substrate via *α*-ketoglutarate to refill the TCA cycle, supporting the robust metabolic switch to glycolysis and assisting in ATP production and biogenesis of citrate and pyruvate (162). Therefore, activated lymphocytes sustain OXPHOS for ATP production, which enhances cell survival and lifespan of lymphocytes and is essential for immune memory (163). Memory T cells also use glucose and other fuels to synthesize triglycerides, which are then used in FAO (163).

Contrary to the dogma that innate immunity is nonspecific and lacks memory, classic innate immune cells such as macrophages, natural killer cells, and monocytes can become epigenetically reprogrammed by infection or vaccination, which confers nonspecific protection from secondary infection, a phenomenon called trained immunity (164). Similar to lymphocytes, there is a metabolic basis to “training” these cells. Specifically, in monocytes stimulated with lipopolysaccharide, a switch from OXPHOS to glycolysis underlies these changes, resulting in increased glucose consumption, lactate production, and NAD^+^/NADH ratio (165). The increase in glycolytic metabolism enables a more robust and swift response to intruding pathogens (166). Training of immune cells is dependent on Akt, mTOR, hypoxia-inducible factor 1*α* (HIF1*α*), and, to a lesser extent, SIRTs. Their crucial roles were affirmed by inhibition of Akt by wortmannin, mTOR by rapamycin, HIF1*α* by ascorbate, and activation of SIRT1 by resveratrol, because these compounds blunt trained immunity (166). Recently, however, the notion that a shift from OXPHOS to glycolysis underlies activation of all immune cells upon microbial stimulation was challenged because pathogen-specific metabolic rewiring was observed in human monocytes. This pathogen specificity was proposed to derive from signaling strength, rather than qualitative signaling differences between microbial stimuli, and consequently mediates different functional outputs such as phagocytic capacity (165).

Adipose tissue macrophages that have been activated and rely on glucose are proinflammatory (type M1) and contribute to adipose inflammation and insulin resistance. Conversely, macrophages that rely on fatty acid metabolism secrete anti-inflammatory cytokines and thus preserve insulin sensitivity of liver and adipose tissue (type M2) (167, 168). Proinflammatory activation can be achieved by overexpression of GLUT1, even in the absence of other conventional stimuli, or by decreasing expression of lipid trafficking proteins, such as fatty acid transport protein 1 (FATP1). FATP1 knockout mice fed high-fat diets showed an increased proinflammatory phenotype and worsened metabolic syndrome than mice with normal FATP1 expression. Alternatively, overexpression of FATP1 decreased substrate switching to glucose and reduced inflammation (169). Thus, macrophage inflammatory status is mediated by rerouting metabolic pathways.

The metabolic switch of glucose metabolism generates ROS that drive the production of inflammatory enzymes, cytokines, and chemokines such as IL-6, monocyte chemoattractant protein-1, TNF-*α*, and inducible NO synthase (iNOS). iNOS is an important metabolic regulator of the immune response because NO inhibits OXPHOS and oxidative metabolism, thus promoting the glycolytic and proinflammatory phenotype (169, 170). In this way, low-grade systemic inflammation (defined as a twofold to threefold increase of circulating inflammatory mediators) including the infiltration of immune cells, particularly in metabolic tissues that have reached their capacity limits, can be driven by metabolic inflexibility (158). Recently, inhibition of iNOS in mouse macrophages was shown to dampen the M1 phenotype through reduction of NO-induced OXPHOS inhibition and assist in the phenotypic and metabolic M1 to M2 repolarization, suggesting that editing macrophage (re)polarization is a promising target to reduce inflammation and promote tissue repair (170).

“Metabolic inflexibility is correlated to an increased risk of certain types of cancers.”

An example of metabolic inflexibility and disrupted inflammatory assuagement is sepsis. During sepsis, a profound change in acute leukocyte metabolism occurs. Metabolic inflexibility drives sepsis-related innate immunoparalysis as the metabolism through glycolysis, *β*-oxidation, and OXPHOS pathways in leukocytes is downregulated, resulting in their inability to mount any response whatsoever (171). A sudden mitochondrial complex I dysfunction in sepsis (172), possibly linked to the overproduction of NO and ROS, may be one of the causes of an upstream mitochondrial gridlock, and has been observed to relate to organ dysfunction (172). Moreover, the impaired metabolic rate has been associated with reduced levels of mtDNA and mRNA expression of OXPHOS components (173, 174). In summary, metabolic flexibility is not only necessary to mount an adequate immune response but also for mitigation of the inflammatory process.

### Cardiovascular disease

Cardiac performance is sustained by fatty acid and glucose oxidation, although fatty acids are the preferred substrate in the heart because of the higher energy yield compared with glucose. This flux is mediated by a high expression of PPAR*α*-regulated genes encoding key proteins in fatty acid uptake, esterification, and oxidation (175). Under energetically demanding conditions such as exercise, the heart switches to the oxidation of glucose and lactate (176). An increase in heart rate increases mitochondrial calcium concentration (177), allowing higher mitochondrial ATP production rates to sustain the increased energetic load of the heart. Upon exercise-induced sympathetic nervous system stimulation, *β*-adrenergic signaling increases glycolytic flux via cAMP activation of cAMP-dependent protein kinase A, increasing pyruvate production and glucose metabolism. Protein kinase A also activates phosphofructokinase-1 and PDH, stimulating the heart to rapidly oxidize glucose even in the presence of fatty acids (178). As a consequence, triglyceride accumulation in cardiomyocytes likely leads to abnormal lipid signaling, increased ROS production, ER stress, and mitochondrial dysfunction (179). Glucose metabolism is enhanced in a similar manner through insulin and nutrient stress signaling via Akt and AMPK, respectively (178).

A dependency on glucose (and ketone body) metabolism is also observed in myocardial ischemia, ventricular hypertrophy, and systemic hypertension (179, 180), as is mitochondrial dysfunction (181, 182). A recent study in mice demonstrated that mildly increasing PPAR*α* expression in the progressive phase of heart failure, when FAO is decreased, maintains myocardial function and energetics, suggesting that modulating substrate utilization may be a promising therapeutic strategy for heart failure (175).

Obesity can cause metabolic inflexibility of the heart and alter substrate selection (179). High-fat diet feeding and consequent insulin insensitivity, for instance, are known to cause cardiac metabolic inflexibility and reliance on fatty acids for energy production through PDK4 inhibition of PDH. Similar to T2DM, increased circulating fatty acids only exacerbates the feed-forward dependency on fatty acid substrates for energy production through the allosteric inhibition of enzymes involved in glycolysis (25). Conversely, the failing heart becomes metabolically inflexible with a decreased capacity to use fatty acids and an increased dependence on glucose metabolism (175). The switch from fatty acid preference to glucose is maintained by increased acetyl-CoA production from pyruvate and subsequent increases in malonyl-CoA concentration, which inhibits CPT-1 and thus FAO (179).

### Cancer

Metabolic inflexibility is correlated to an increased risk of certain types of cancers (183) and metabolic syndrome is associated with a 33% elevated total cancer mortality (184). Epidemiological evidence shows that through their relation to insulin resistance, excess body weight, and T2DM are associated with an increased risk of pancreatic, liver, and endometrial cancers, among others, and of colon cancer in males (185). Excess body weight increases the risk of cancer via augmented circulating levels of leptin and decreased circulating levels of adiponectin (185). Physical exercise is inversely related to certain types of cancers and may reduce the risk of cancer by changing insulin and IGF and/or sex hormone levels, decreasing body weight and positively modulating immune function (186).

Diet composition is also correlated to development of certain cancers [reviewed in Potter *et al.*^187^)]. High-fat diets for instance have particularly been related to increased risk of colorectal (188), pancreatic (189), breast (190), lung (191), and prostate cancer (192). Recently, tumor cell metastasis in mice was found to be under strong control of the tumor cells’ potential to express the fatty acid receptor CD36 and metabolize fatty acids. Additionally, dietary fatty acid exposure increased tumor cell expression of CD36 and increased metastasis in mice (193). Besides diets with a high fatty acid content, diets with a high amount of animal-derived amino acids also increase the risk of cancer in the middle-aged human population (194). Caloric restriction, however, is known to reduce cancer occurrence (191) (see “Prolonged fasting and caloric/dietary restriction”). Rodent studies have shown that CR alters insulin and IGF-1 levels via a reduction in phosphatidylinositol 3-kinase (PI3K)/Akt signaling. Moreover, inhibition of mTOR, which is downstream of PI3K/Akt, by prolonged rapamycin treatment, delays cancer formation in aged mice (195). Reducing carbohydrate intake reduced tumor growth in mice (196). In rhesus monkeys, CR reduced the incidence of neoplasia by 50% (197). Currently, clinical studies are under way, but various human studies point toward a reduced incidence of cancer after caloric restriction (198).

#### Tumor growth and metabolic flexibility

Our understanding of cancer metabolism has rapidly advanced in recent years. Most cancer cells show a remarkable metabolic flexibility, which allows a survival advantage in the face of their energetic demand and the environmental supply of nutrients. Mitochondrial-mediated flexibility is central in this process [reviewed in Vyas *et al.* (199)]. Metabolic adaptations that underlie clonal evolution of tumor cells to a metastatic phenotype suggest that tumor cells do not become metabolically hardwired but remain able to reroute metabolism to adapt to their phenotype and the newly acquired environment (200). Indeed, reducing metabolic flexibility in cancer cells may lead to potential treatment options, because metabolic interference can come at a substantial cost to oncogenic potential (1).

Tumors and their environment can be very diverse and, as such, their metabolism and substrate preference is also diverse (201). Common traits, however, include increased glucose consumption via glycolysis and enhanced glutamine metabolism to support the energetic and anabolic demands of proliferation. Notwithstanding the diversity of cancers, malignant cells share a common metabolic trait, namely that they can acquire and use nutrients from a predominantly nutrient-poor environment, a *modus operandi* that emerged as a promising target to battle tumors (202–205). Most tumors display one, if not several, cancer associated metabolic hallmarks: (1) deregulated uptake of glucose and amino acids, (2) use of opportunistic modes of nutrient acquisition, (3) use of glycolysis/TCA cycle intermediates for biosynthesis and reduced nicotinamide adenine dinucleotide phosphate (NADPH) production, (4) increased demand for nitrogen, *(*5) alterations in metabolite-driven gene regulation, and (6) specialized metabolic interactions with the microenvironment (206). As a prominent feature of cell activation and proliferation, tumor cells chiefly require increased amounts of glucose and glutamine to survive (207).

The metabolic reprogramming that underlies increased glucose consumption for use in glycolysis, as opposed to OXPHOS, is known as the Warburg effect. In 1924, Otto Warburg discovered that cancer cells metabolize glucose differently than cells of normal tissues: that even in conditions of sufficient oxygen availability, cancer cells convert glucose into lactate instead of using glucose for OXPHOS (208). Warburg hypothesized that cancer cells have mitochondrial defects and impaired aerobic respiration that forces them to rely on glycolysis. Today, we understand that mitochondrial respiration is not impaired but that cancer cells place emphasis on acquisition and generation of building blocks necessary for cell division. They do so by enhancing biosynthetic metabolism using glycolytic intermediates (209). Increased glucose uptake and metabolism is very often mediated by the oncogenic signaling protein Ras, which activates the PI3K/Akt pathway. PI3K/Akt signaling increases the expression of GLUT1 and its translocation to the cell surface, and enhances HK and phosphofructokinase activity, thus capturing glucose and increasing glycolytic flux (196). Interestingly, glucose catabolism in cancer cells is partially uncoupled from the TCA cycle and OXPHOS because increased activity of PDK dampens glucose metabolism through negative feedback on PDH (210, 211). A glycolytic switch involving differential expression of pyruvate kinase isoforms and stabilization of HIF1*α* upregulates rate-limiting enzymes within branching pathways of glycolysis, ensuring that glycolytic intermediates are free to take part in diverse biosynthetic reactions that are essential for increased proliferation (196). These alternative pathways include the PPP using glucose-6-phosphate, hexosamine biosynthesis using fructose-6-phosphate, phospholipid biosynthesis using dihydroxyacetone phosphate, and glycine and serine biosynthesis using 3-phosphoglycerate. The PPP is chiefly used for NADPH and ribose synthesis to produce nucleotides (206). Of particular significance is serine production because it is a major substrate for the folate/one-carbon cycle whose metabolites contribute to various cellular biosynthetic and regulatory processes (212, 213). Lactate, produced from glycolysis, is excreted from the cell or used in biosynthetic reactions such as aspartate synthesis (1). Aspartate is used to support protein and nucleotide synthesis in proliferating cells and sustains proliferation in the face of OXPHOS impairment (214). Even TCA cycle intermediates are not solely used to produce NADH for mitochondrial respiration, but intermediates can also be used to form nonessential amino acids and fatty acids, which facilitate protein synthesis, membrane construction and cholesterol synthesis (206).

Glutamine is an essential nutrient for cancer proliferation and its dependency is also referred to as “glutamine addiction” (215). Glutamine can either be used as an important anaplerotic substrate in the TCA cycle, a carbon and nitrogen donor, or for production of purine and pyrimidine nucleotides that are necessary for DNA replication (216). Intracellular glutamine can also be used as a substrate for the large neutral amino acid antiporter (LAT1). LAT1 can couple glutamine export with import of essential amino acids. Compared with glucose, glutamine tumorigenesis-associated metabolic reprogramming is only recently becoming clear. In proliferating cells, the transcription factor Myc is a major driver of glutamine utilization and is frequently targeted for upregulation in various tumors, despite the abundance of glucose. This glutamine addiction is beneficial for the cancer cell because it maintains mitochondrial TCA cycle integrity and provides the cell with large quantities of NADPH needed to meet the demands of cell proliferation (215). Besides Myc-regulated glutamine addiction, the activity of the Rb tumor suppressor protein family, which negatively regulates glutamine uptake, is reduced, facilitating increased uptake of glutamine. However, not all tumors are glutamine dependent because some tumors and embryonic stem cells are capable of proliferation without an exogenous supply of glutamine, because they can synthesize it (206).

“Some tumors and embryonic stem cells are capable of proliferation without an exogenous supply of glutamine.”

## Interventions to Improve Metabolic Flexibility

### Lifestyle

Lifestyle interventions are pertinent for patients with metabolic syndrome. Most patients with T2DM are overweight or obese and do not exercise frequently. Interventions that reduce body weight by as little as 5%, however, can reduce obesity-related metabolic disorders (217). In particular, loss of visceral adipose tissue is related to improved metabolic control of fasting glucose, cholesterol/high-density lipoprotein ratio, triglycerides, and diastolic blood pressure (218). Lifestyle interventions to reduce body weight predominantly include exercise training and controlled reduced caloric intake, but their efficacy depends on age, sex, ethnicity, and body weight upon inclusion (219). As such, caution must be taken when interpreting results when assessing metabolic flexibility using suboptimal methods, because individual variability and experimental setup can considerably influence results.

#### Exercise training

Physical inactivity is likely one of the primary causes of metabolic inflexibility (220, 221); regular habitual physical exercise has long been known to increase metabolic flexibility (7). As such, exercise training regimens can be used as an intervention to improve metabolic flexibility. Generally, exercise can be divided into two extremes: aerobic (or endurance-based) and anaerobic/glycolytic (resistance-based) activities. Both promote considerable health benefits such as increased mitochondrial content and improvements in glycemic control (222). For example, a 10-day endurance exercise training regimen increases FAO in the absence of increased mitochondrial content. A high-intensity exercise training program, however, showed elevated citrate synthase and *β*-hydroxyacyl CoA dehydrogenase activity after 5 days and increased levels of mitochondrial complexes after 10 days (51).

AMPK is an important regulator of exercise-induced effects on metabolic flexibility (223). Acute AMPK activation reduces glycogen and protein synthesis while promoting glucose transport and FAO (222). A higher mitochondrial volume density and improved mitochondrial quality can be seen as consequences of chronic activation of AMPK and expression induction of PGC1*α*, myocyte-specific enhancer factor 2 (MEF2), NRF-1 and NRF-2, and nuclear expulsion of histone deacetylase 4 and 5 (41, 222, 224). Contraction-induced calcium uptake acutely increases OXPHOS (94, 177) and augments glucose transport and stimulates lipid uptake and oxidation through MEF2-induced expression of GLUT4 and PGC1*α*, respectively. Additionally, PGC1*α* is expressed upon muscle contraction-induced activation of p38 MAPK (222). Endurance exercise increases the activity of muscle oxidative enzymes and FAO, in part from increased volume of the mitochondrial reticulum and elevated levels of cardiolipin, a lipid that is necessary for the assembly of OXPHOS complexes (41, 51, 225).

Regular physical exercise positively influences insulin-stimulated glucose uptake and mitochondrial function in skeletal muscle, and, importantly, in patients with T2DM (41). FAO in skeletal muscle increases during physical exertion independent of body mass index, although regular exercise is likely needed to sustain a long-lasting impact on metabolic flexibility (10). Particularly combined with weight loss, exercise training improves insulin sensitivity, mitochondrial content, and fasting FAO (220). Intriguingly, type II, glycolytic, muscle fiber density is higher in obese and insulin-resistant patients, although it is unknown whether these are due to inactivity or impaired glucose metabolism (226). Observations of reduced PGC1*α*, AMPK (227), and mitofusin-2 (151) expression in insulin-resistant individuals after exercise might provide mechanistic information as to why mitochondrial function improves more in healthy volunteers compared with patients with T2DM and obesity.

Although skeletal muscle is considered the main site at which the adaptive responses of exercise on metabolic flexibility occur, it is becoming increasingly evident that other tissues are also involved (see “Physical exercise”). Regular exercise can, for instance, reduce adipose cell size and enhance adipose glucose metabolism, resulting in improved insulin sensitivity in both adipose and muscle tissue. Moreover, habitual physical exercise remodels subcutaneous adipose tissue by stimulating browning in mice (228). In rats, chronic endurance exercise induces browning in subcutaneous WAT concomitant with increased mobilization of energy stores, which were attenuated in animals fed high-fat diets. The browning program initiated by exercise training promoted expression of PPAR*α* and PPAR*γ*, AMPK, PGC1*α*, and adipose triglyceride lipase (229). Although the exact mechanisms underlying this beneficial effect are still under investigation, exercise training in humans reduced intrahepatic lipid content (230). In mice, PGC1*α* is required for an exercise-induced increase in mitochondrial volume density and reduction in intrahepatic lipid content (231). Also, in the human heart, exercise reduces cardiometabolic risk factors by increasing insulin sensitivity, decreasing cardiac lipid content, and improving glucose tolerance (232). In mice, exercise increased cardiac PGC1*α*, NRF1, and TFAM expression and augmented mitochondrial volume and number, which were all dependent on endothelial nitric oxide synthase (232).

Interestingly, exercise training also drives metabolic adaptations through epigenetic mechanisms. Short-term, high-intensity exercise decreases muscle promoter methylation of genes involved in mitochondrial function such as PGC1*α*, TFAM, MEF2A, and PDK4, whereas in patients with T2DM, these regions usually have higher methylation levels (137). High-fat feeding in mice induced PGC1*α* hypermethylation that was transferable to the offspring. Maternal exercise, on the other hand, prevented high-fat feeding hypermethylation of PGC1*α* and mitigated epigenetic associated metabolic dysfunction in the offspring (138). Indeed, moderate exercise during pregnancy in humans is advised because it reduces the risk for obesity during the progeny’s childhood and preadolescence (138). Although more research is necessary, it is clear that regular exercise and exercise training may aid in reversing the pandemic of metabolic disease.

#### Dietary interventions

Weight loss is an important step in restoring metabolic flexibility and is the most common intervention for obesity and obesity-related metabolic comorbidities. Generally, energy-restricting diets are aimed at inducing a state of negative energy balance so that stored lipids inside adipocytes are used as alternative substrates (233). Energy-restricting dietary regimens have proven effective in augmenting metabolic flexibility in animal studies and hold promise for application in humans (234). During intermittent fasting, subjects go for extended periods with little or no energy intake, with intervening periods of normal energy intake. Intermittent fasting in rodents improved insulin and leptin sensitivity increases ketone body levels and reduced adiposity and inflammation (235). CR without hunger- or disease-related malnutrition in animals and humans results in healthier aging through improved metabolic health, reduced obesity, and the risk of T2DM, cancer, and cardiovascular disease (236). Additionally, CR is considered the most robust intervention to increase longevity in animal model systems and ameliorate age-associated disease in primates and humans (see “Prolonged fasting and caloric/dietary restriction” and “Aging”) (38).

Maintaining an energy-restricting diet, however, is challenging, because most people have difficulties maintaining compliance over long periods. Moreover, a recent study highlighted the paucity of clinical evidence supporting energy-restricting diets in humans (237). Although weight loss is generally achieved in overweight and obese subjects, potential adverse effects exist for leaner subjects (237). Recommendations such as interruption of sedentary behavior with repeated low-intensity exercise, as well as “exercise snacking” regimens wherein brief vigorous exercise is followed by meal consumption, may prove more effective than the standard recommendation to “eat less and move more” (4). Because compliance to energy-restricting diets is challenging, interventions that alter meal timing without reducing total caloric intake are actively pursued. Popular concepts such as increasing or decreasing meal frequency, however, lack concrete scientific evidence supporting their efficacy (238).

Recently, attention has arisen for food intake restricted to the active time phase (239). Alterations in feeding patterns outside the active phase can disrupt the synchrony between the central and peripheral clocks and disturb metabolic flexibility (see “Interplay between circadian rhythm and metabolic flexibility”). In rodents, food intake outside the active phase causes obesity, whereas time-restricted feeding protects against obesity and insulin resistance (240). Time-restricted feeding restores both cycling of metabolic regulators such as cAMP response element-binding protein, mTOR and AMPK, and circadian clock gene expression (214).

Besides dietary interventions to reduce overall energy intake or restrict energy intake to restricted periods, specific dietary constituents can induce changes in metabolic flexibility. For instance, carnitine is closely associated with the mechanism of metabolic flexibility (241). Carnitine plays a role in the import of long-chain fatty acids into the mitochondria for use in *β*-oxidation and in the mitochondrial efflux of excess carbons in the form of acyl-carnitines (242). Mechanistically, during substrate opulence or a deficiency of carnitine or carnitine acetyltransferase, accumulation of acetyl-CoA in skeletal muscle allosterically inhibits PDH resulting in impaired glucose utilization and whole-body glucose tolerance (243). In obese rats, free carnitine in skeletal muscle is decreased and supplementation of l-carnitine restored metabolic flexibility (242). In patients with T2DM, carnitine acetyltransferase expression is severely perturbed and free carnitine concentrations in diabetic mice are decreased compared with controls (243, 244). Although not yet in clinical practice, l-carnitine supplementation improves metabolic flexibility by decreasing plasma glucose and insulin levels and increasing PDH activity in muscle of insulin-resistant subjects (243). Carnitine metabolism may also be involved in the regulation of mitochondrial protein acetylation, because acetyl-CoA serve as acetyl donors and protein hyperacetylation is observed in high-fat feeding of mice (151).

“Exercise training regimens can be used as an intervention to improve metabolic flexibility.”

### Pharmaceuticals

**Figure 5.**
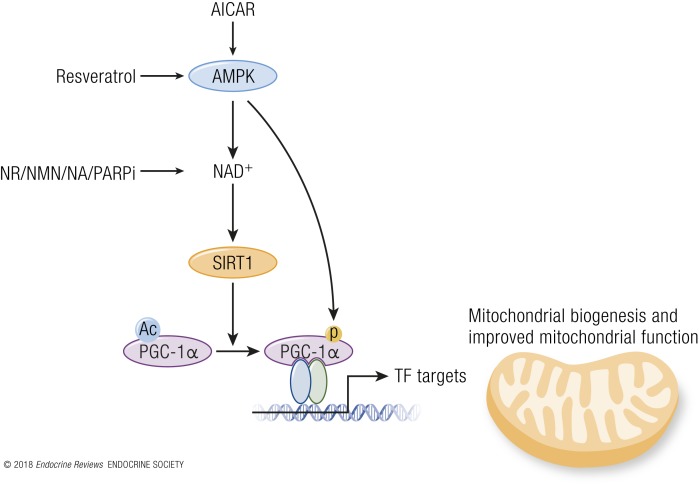
A selection of pharmaceutical compounds that target major players or key nodes in metabolic circuits, such as AMPK and sirtuins. Via altered transcription factors, these compounds act on mitochondrial function and positively affect metabolic flexibility. See “Pharmaceuticals” for a brief description of the examples mentioned here. Ac, acetyl; NA, nicotinic acid; NMN, nicotinamide mononucleotide; NR, nicotinamide riboside; P, phosphate; PARPi, poly (ADP-ribose) polymerase inhibitor; TF, transcription factor.

Pharmaceutical approaches to improve metabolic flexibility have been studied in great detail. Most pharmaceutical therapeutics target major players or key nodes in metabolic circuits, many acting on mitochondrial function (245). The examples that follow provide strong support for continuing the search for future pharmacological principles that enhance metabolic flexibility (Fig. 5). For more details on the mechanisms underlying the beneficial effects of these treatments on metabolic flexibility, we refer the reader to some excellent reviews described in each topic.

#### AMPK

AMPK is activated by a high intracellular AMP/ATP ratio and activates transcription factors, such as the FOXO proteins, to increase mitochondrial energy production. Metformin is a biguanide and reduces hepatic glucose production and increases insulin sensitivity by activating AMPK, although several AMPK-independent mechanisms have been proposed [reviewed in Pryor and Cabreiro (246)]. Metformin is one of the first-line treatments of patients with T2DM, but it has also been used to treat patients at risk for T2DM, such as those with metabolic syndrome (87).

Resveratrol (3,5,4′-trihydroxystilbene) is a plant-derived polyphenol that activates AMPK and, via SIRT1, increases PGC1*α*, PGC1*α* deacetylation, mitochondrial size and density, and mtDNA content in skeletal muscle [reviewed in de Ligt *et al.* (247)]. Resveratrol treatment also increased physical endurance and protected from high-fat diet-induced muscle accumulation of diacylglyceride and ceramide, and related mitochondrial dysfunction (248). Because resveratrol activates mitochondrial biogenesis through the AMPK-SIRT1-PGC1*α* axis, it prompts mitochondrial biogenesis, the unfolded protein response, and autophagy machinery that are known to extend longevity in animals (37, 249). Animal studies have also demonstrated that resveratrol could stimulate energy expenditure and protect against a high-fat diet-induced weight gain (250), via induction of FAO and reduced lipogenesis, mediated by activation of the AMPK-SIRT1 axis (251, 252). In the context of insulin-controlled metabolic flexibility, rodent studies largely show improved insulin sensitivity and glucose tolerance in models of obesity, diabetes, and metabolic dysfunction [reviewed in de Ligt *et al.* (247)]. Clinical studies in humans suggest that resveratrol may improve insulin sensitivity and reduce plasma levels of glucose and insulin in patients with T2DM and mimic CR in obese subjects (253, 254). As such, resveratrol use by humans is particularly beneficial in reversing the early stages of metabolic disorders. Full confirmation of these beneficial effects in humans by placebo-controlled clinical trials remains relatively limited. Variation in duration and dose of resveratrol may explain the diverse outcomes of these studies (247).

The AMPK agonist 5-aminoimidazole-4-carboxamide riboside (AICAR) improves skeletal muscle glucose uptake and transport, fatty acid uptake, mitochondrial protein content, and insulin sensitivity in mice (223). AICAR also rescued mitochondrial function in mice deficient in cytochrome *c* oxidase and augmented exercise endurance in healthy animals in a PGC1*α*-dependent manner, even if they were untrained (255, 256). Chronic exposure of AICAR reduced white adiposity and increased OXPHOS in rat hearts by increasing PGC1*α* expression and FAO (229), as well as glucose uptake (257). Figure 5 summarizes the potential role of AMPK activators for metabolic flexibility.

#### SIRTs

Through SIRTss, NAD^+^ provides a direct link between the cellular energy state of the cell and control of signaling and transcriptional events [reviewed in Houtkooper *et al.* (258)]. The SIRT family of NAD^+^-dependent deacylases, comprising seven members that vary in tissue specificity, subcellular localization, enzymatic activity, and targets, mediates metabolic flexibility in animal models (42). SIRT1 and SIRT3 have particularly received attention in this respect. SIRT1 is predominantly found in the nucleus, although it can also be found in the cytosol. SIRT1 controls the activity of transcription factors and cofactors such as p53, MEF2, FOXO, and PGC1*α*, which govern mitochondrial biogenesis and activity and lipid and glucose metabolism (42). SIRT3, which is localized in the mitochondrial matrix, targets many proteins involved in metabolic homeostasis, including OXPHOS subunits (42). Acetylation of mitochondrial proteins propagates metabolic inflexibility and deacetylation promotes metabolic flexibility (178, 259).

NAD^+^ levels can be increased by supplying biosynthetic precursors, intermediates, or by inhibiting NAD^+^ consuming enzymes [reviewed in Carafa *et al.*^260^)]. Nicotinamide riboside, nicotinamide mononucleotide, and nicotinic acid are pursued as strategies to boost metabolic flexibility through increased NAD^+^ and consequent activation of SIRTs (261, 262) (Fig. 5). Boosting NAD^+^ levels has also been investigated as a strategy to increase lifespan as NAD^+^ levels decline during aging in model systems and humans (263). For instance, nicotinamide riboside, which is converted to nicotinamide mononucleotide via the NAD^+^ salvage pathway in eukaryote cells, can boost NAD^+^ synthesis and can enhance oxidative metabolism and protect mouse tissues from high-fat diet-induced metabolic abnormalities (264) by means of PPAR*γ* and antioxidant gene upregulation (265). Additionally, nicotinamide riboside treatment in aging mice increased skeletal muscle function by preventing stem cell senescence, improved mitochondrial function, and a higher expression of genes involved in the TCA cycle and OXPHOS (266). Nicotinamide mononucleotide conversion to NAD^+^ activates SIRT1 and improves glucose homeostasis in mice (42). Nicotinamide mononucleotide administration to mice also enhances energy metabolism, promotes physical activity, improves lipid profiles, and ameliorates age-related pathophysiology (267). Alternatively, pharmacological inhibition of the NAD^+^ consuming enzyme poly (ADP-ribose) polymerase in mice activates SIRT1 and increases mitochondrial respiratory capacity leading to augmented fitness and protection from diet-induced obesity (268). Moreover, poly (ADP-ribose) polymerase inhibition rescued mitochondrial respiration defects and increased FAO in myotubes from obese patients by augmenting mitochondrial function (268).

#### PPARs

PPARs are lipid sensors that transcriptionally modulate metabolic programs in response to nutrition and are interesting drug targets to improve metabolic flexibility [reviewed in Bugge and Holst (269)]. There are three PPAR isotypes (PAR*α*, PPAR*β*/*δ*, and PPAR*γ*), which differ in ligand affinity and tissue distribution [reviewed in Gross *et al.* (270)]. Fibrates activate PPAR*α* and are commonly used for treatment of hyperlipidemia. Of the fibrate drug class, fenofibrate and bezafibrate have recently gained interest as interventions to improve metabolic flexibility, in particular for the treatment of insulin resistance. Fenofibrate improves FAO in primary human skeletal muscle cell cultures from obese and insulin resistant subjects. *In vitro* and in animal models, PPAR*α* activation increased expression of PDK and CPT1 (142). In insulin-deficient mice, bezafibrate improves impaired glucose metabolism by augmenting hepatic mitochondrial performance [reviewed in Komen and Thorburn (255)], suppressing hepatic inflammatory pathways, and improving insulin sensitivity (271). Similarly, synthetic PPAR*β*/*δ* agonists such as GW501516 or L-165041 reduce adiposity improve insulin sensitivity and hyperlipidemia in obese patients, through their ability to augment mitochondrial biogenesis and activity (249). PPAR*β*/*δ* agonists also enhance energy expenditure by increasing fatty acid catabolism in adipose tissue and skeletal muscle (270). Another PPAR agonist is tesaglitazar, which binds and activates PPAR*α* and PPAR*γ*. Tesaglitazar increases whole-body glucose metabolic insulin action in obese rats by reducing hepatic glucose output, restoring skeletal muscle glucose uptake, and suppressing free fatty acid release by adipocytes (272). Finally, thiazolidinediones, such as rosiglitazone, potently activate PPAR*γ* and lower blood glucose levels in patients with T2DM. Thiazolidinediones improve skeletal muscle glucose disposal via upregulation of GLUT1, decrease liver glucose output, and, as a primary target, improve the lipid-buffering capacity of WAT and as such improve hepatic steatosis (270, 273). However, thiazolidinediones were rapidly sidelined as therapeutics because of their potential adverse effects such as an increased risk of myocardial infarction (274).

#### mTOR

mTOR is a central regulator of growth and metabolism in all eukaryotes and its activity depends on the cells energy and nutrient levels [reviewed in Laplante and Sabatini (275)]. In general, anabolic processes such as protein and lipid synthesis, and protein turnover are controlled by mTOR complex 1 (mTORC1). As such, mTORC1 controls the balance between anabolism and catabolism in response to changing environments. mTORC1 facilitates cellular growth by a shift in glucose metabolism from OXPHOS to glycolysis, likely through an mTORC1-mediated increase in the translation of the transcription factor HIF1*α* and an increased flux through the PPP, which uses carbons from glucose to generate NADPH and other intermediary metabolites needed for proliferation and growth (276). A reduction in cellular energy charge, such as during glucose deprivation or fasting, inhibits mTORC1 by AMPK-dependent as well as AMPK-independent pathways, and is required for the generation of ketone bodies in the liver (277). Similarly, low levels of amino acids (particularly arginine and leucine) and interaction with AMPK inhibit mTORC1 activity. In these ways, energy-consuming processes such as mRNA translation are inhibited during periods of low energy.

mTORC2 instead mainly controls cellular proliferation and survival, and has more limited effects on metabolic flexibility *per se*. What should be noted, however, is that mTORC2 directly activates Akt downstream of insulin/PI3K signaling; as such, mTORC2 inhibition disrupts the physiological response to insulin.

Tissue-specific mTOR signaling can have profound effects on whole-body metabolism. For instance, increased mTOR signaling in either adipose tissue, skeletal muscle, or the liver, negatively affects systemic glucose and insulin homeostasis (278). Indeed, mTOR signaling is dysregulated in cancer, T2DM and obesity, and is therefore actively perused as a promising drug target [reviewed in Laplante and Sabatini (275 and Albert and Hall (278)].

One compound that particularly received ample attention as an inhibitor of mTOR is rapamycin. Rapamycin is a natural bacterial product that acutely inhibits mTORC1 and, after prolonged treatment, also inhibits mTORC2 in some cell types. As such, rapamycin has both positive and negative effects on metabolism depending on dose and duration. Short-term rapamycin treatment in mice instigates glucose intolerance, insulin resistance, and immunodeficiency. Prolonged treatment improves metabolic profiles, increases oxygen consumption and ketogenesis, and markedly enhances insulin sensitivity [reviewed in Li *et al.* (279)]. Additionally, rapamycin has earned great interest over the years to prolong lifespan (195, 280), even when administered to aged animals (281). Rapamycin, however, has poor solubility and pharmacokinetics, which led to the production of rapamycin analogs (rapalogs), which are currently used in some cancer therapies (279).

Manipulating mTOR signaling is, however, very complex, because many positive and negative feedback loops exist, reducing efficacy of mTOR targeting compounds. Moreover, because mTOR controls many vital cellular processes, its complete inhibition by high doses of rapamycin may negatively affect the maintenance of tissue functions and provoke adverse events (275). Our molecular understanding of mTOR signaling is not yet comprehensive, and future research into rapalogs with fewer pleiotropic and adverse effects is necessary and will likely lead to therapeutics to ease metabolic disease.

## Future Perspectives

Metabolic flexibility can be studied at various levels, from cell to whole-body studies. However, metabolic flexibility is likely best understood as a system of interacting components (282). As such, metabolic flexibility should be measured by the ability to adapt to conditions of temporary stress, such as physical exercise, infections, or mental stress, in a healthy manner (283).

### How to understand metabolic flexibility from cellular studies

To understand metabolism at the system level, cellular metabolic programming, such as biochemical pathways and networks thereof, should be well annotated first. Metabolic flux analysis, flux balance analysis, and metabolic pathway analysis are among the most popular tools in stoichiometric network analysis and are used extensively to study (cancer) metabolism (284, 285). In general, intracellular fluxes are measured using isotope labeling of substrates, after which isotopomer distribution in metabolites can be quantified (286). For instance, mass-isotopomeric flux analysis of glycolysis and the TCA cycle in a rat-derived *β*-cell line using [U-^13^C]-d-glucose has demonstrated that insulin secretion and oxygen consumption correlate with citrate synthase rates, and pyruvate carboxylase rates showed the highest fold change in response to glucose stimulation, confirming these enzymes as important nodes in glucose metabolism (287). Labeling with [1,2-^13^C]-glucose and [U-^13^C]-glutamine revealed considerable differences in metabolic pathway fluxes between the exponential growth and stationary phase of Chinese hamster ovary cells. Specifically, glycolytic flux and lactate production was high and PPP flux low in the exponential growth phase, which was collectively reversed in the stationary phase concomitant with lactate consumption and reduced TCA cycle flux. Interestingly fatty acid biosynthesis remained high in both growth conditions (288). Recent advances in metabolite extraction from isolated mitochondria demonstrate that metabolic flux models can be built with increasing precision (289). Translation of the collected data, however, will likely be the most challenging task in the future (290). Finally, metabolic flexibility modeling can give us insight into the evolution of metabolic pathways (291), demonstrating that genes encoding enzymes with high connectivity and high metabolic flux have a greater chance of conservation (12) and are the most likely sites of metabolic regulation (292).

### How to understand metabolic flexibility from whole body/animal physiology

The continued use of model organisms to understand metabolic flexibility regulation is vital. Over the past few decades, model organisms such as primates, rodents, flies, nematodes, and yeast have proven their effectiveness in elucidating pathways and regulatory components involved in metabolic flexibility. In humans, mixed meal tests, glucose clamping, isotope administration, and indirect calorimetry are regularly used to assess metabolic flexibility in intervention studies. Scientists have limited their research on a small number of animal models to further understand metabolic flexibility and its regulation. Some examples are discussed in the following sections.

The round worm *Caenorhabditis elegans*, for instance, is used to investigate metabolic pathways and has been instrumental in elucidating key genetic regulators of metabolism in relation to lifespan (36, 293, 294). Inbred lines have been generated for *C. elegans* (295) and, recently, metabolic network models became available (296, 297). With relative ease, dietary and genetic influences can be simultaneously investigated in *C. elegans* (298). Moreover, important advances have been made in quantifying the metabolic spectrum in *C. elegans* (299).

Work in mice and rats has particularly forwarded the field. For example, mice have been the driving force behind research into CR (300), and the use of recombinant inbred lines such as the BXD mouse strains now offer insights into the role of genetic background effects and can help translate the differences between gene variants into meaningful data for human populations and disease. The natural variation in gene expression that is present in the human population is also recapitulated in these models and therefore more honestly reflect genotype-phenotype associations than artificial loss-of-function situations, such as gene knockouts or knock-down experiments (82). For instance, 25% of the BXD strains, which were generated by crossing C57BL/6J and DBA/2J, have a hypersecretion of insulin in response to glucose (301). Moreover, OXPHOS gene expression and supercomplex assembly, and PPAR and cholesterol biosynthesis pathways, which are known to influence metabolic diseases, are highly variable in the BXD population (302).

Although rats and mice share a high metabolic homogeneity with humans, they differ in many aspects. For instance, vitamin C and bile acid synthesis pathways are different in rats and humans (303). Additionally, the discrepancies between sex, or strain variations in sensing and handling of substrates, may convolute the interpretation of data derived from animal model systems (304). Differences in experimental approaches and the techniques used to measure metabolic flexibility may influence outcomes (305). Methodological differences can severely impact interpretation of study data (106), advocating for a gold-standard application of available methods and technology. For instance, rodent models are susceptible to temperature and as such can show divergent metabolism depending on their environment. Moreover, cage-housing density of mice can strongly influence glucose metabolism (306). To standardize and validate phenotyping in mice, standard operating procedures were developed by the Eumorphia program to ensure that test outcomes are comparable between different laboratories (307). Finally, many different mechanisms influence metabolism, making the identification of causal mechanisms of metabolic inflexibility challenging. Therefore, high-quality measurements of multiple complementing layers of data including genomics, transcriptomics, proteomics, metabolomics, and phenomics are needed. Such a multilayered cross-omics approach can help elucidate complex traits associated with metabolic flexibility (302).

### Systems biology, mathematical modeling, and stoichiometric network analysis

Through development of mathematical and computational models, systems biology can identify meaningful components and interactions, especially at critical nodes in the network. As such, systems biological approaches can represent, analyze, and predict the behavior of the whole system (158). A good example of this is the global human metabolic network, Recon 1, which was released in 1997 (308). A community-driven reconstruction, Recon 2, was released in 2013 and included 65 cell type–specific models (309). In 2016, Recon was updated as Recon 2.2 and includes a total of 5324 metabolites, 7785 reactions, and 1675 associated genes. Among other topics, Recon is used to predict biomarkers for inborn errors of metabolism, to identify drug targets and possible side effects, and to study cancer metabolism and the interactions between microorganisms and their host (310). Although the models are not yet all-inclusive, they can yield meaningful insights into the regulation of metabolic flexibility. For instance, genome-scale models of cancer metabolism can help us comprehend the complexity of human cancer heterogeneity in relation to substrate preference and therefore expedite the discovery of putative anticancer targets (285). Genome-scale metabolic models can also be applied to investigate genetic variation in genotype-phenotype interactions and as such can be used to identify metabolic biomarkers and discover treatments based on genetic markers (311).

Recently, a compartmentalized mathematical model of human metabolism, which was not based on Recon 2, was implemented to study the effects of insulin resistance on individual tissues (312). The model describes the transport, storage, and utilization of glucose and fats in the human body and suggested that insulin resistance in one tissue created a knock-on effect in other tissues that tries to compensate for the reduced flexibility. The model also simulated that insulin resistance causes fatty liver regardless of the site of insulin resistance in the body, and that slightly reduced skeletal muscle metabolic flexibility is caused by insulin resistance of individual tissues, although the strongest effect on muscle metabolic flexibility is observed when the whole body is insulin resistant (312). At the organ level, flux balance computational modeling of muscle metabolism during the fasted to fed state transition showed that key metabolic phenotypes characteristic of human insulin resistance can be recapitulated by decreasing flux through the PDH complex (313). Notably, these observations corroborate knowledge that competition between fatty acids and glucose occurs at the level of the pyruvate dehydrogenase complex (314). Additionally, maximum glycogen accumulation and homeostatic energy demand were found among the most important parameters regulating muscle cell metabolism (315), which supports previous findings that improving glycogen synthesis strongly influences exercise-induced skeletal muscle insulin sensitivity (227). Collectively, these advances enable us to understand how biochemical networks behave under different conditions and help us identify important actors that can be targeted to eliminate the undesired effects of environmental or inherent factors (284).

“The continued use of model organisms to understand metabolic flexibility regulation is vital.”

### Medical horizons and translational aspects

The wealth of available preclinical data suggests that metabolic flexibility is the cornerstone of many (patho)biological processes. It is this wealth, however, that hampers translation to applicable human interventions. Metabolic flexibility occurs at the substrate, cell, tissue, organ, and organism level and is affected by disease, diet, body composition, and many other (epigenetic) factors.

To gain further insights into our understanding of metabolic flexibility, a coherent, multidisciplinary approach is warranted. Such an approach includes specialists in phenomics, metabolomics, proteomics, genomics, enzymology, molecular biology, bioinformatics, systems biology, clinical genetics, nutrition, whole-cell systems, model systems, and *in vivo* human studies. The latter are particularly required to study the metabolically flexible processes to prevent merely superficial and associative data. However, recent studies show that personalized analysis and tailoring of metabolism open up therapeutic possibilities (316–318). Supporting this collaboration, current diagnosis and research tools need to be improved, for instance in the field of metabolomics, in which faster and more accurate analyses are needed. Major advances can be made by combining standalone analyses into combined methods and applying targeted metabolite platforms that cover emerging groups of metabolites, with a primary focus on human translation.

## Conclusion

Metabolic flexibility is likely best understood as a system of interacting components to manage energy resources and requirements in health and disease (289). Together with the endocrine system, mitochondria orchestrate the metabolic reprogramming necessary to sustain cell function in both healthy and pathological conditions.

Metabolic flexibility has a broad scope that can be daunting; nevertheless great progress has been made over the past few decades to understand its underpinnings (107). However, because of the complexity of metabolic flexibility, many ends remain untied and solid translational steps are often missing. The use of isotope labeling to determine metabolic flux and substrate preference is a common practice (319). Flux measurements can aptly portray the dynamics of metabolism, whereas protein and gene expression analyses are typically static snapshots in time. A biochemical revival of flux metabolism research will surely reveal insights into metabolic flexibility.

Unveiling the key denominators of metabolic flexibility as targets for intervention strategies is paramount to stop the rise of metabolic disease. Besides abating metabolic inflexibility such strategies may even prolong health span and lifespan. To advance translational research from bench to bedside, clinical trials need to be performed that are well-controlled and use state-of-the-art methods to measure metabolic flexibility.

With this review, we have summarized the current status of metabolic flexibility research and realize that, because of the vast amount of literature and the complexity of this research field, it is not comprehensive. Nonetheless, it is evident that metabolic flexibility can be placed in the broad context of health and disease and a deeper understanding of its intricacies will significantly affect health care. As a final note, the situation portrayed here does not necessarily reflect that of each individual. Because of the genetic and epigenetic disparity of humans and the enormous varieties in lifestyle, it is not unthinkable that each person fashions a unique way to maintain energy homeostasis. In light of the rapid developments in the field of nutrigenomics and personalized medicine, future research will likely focus on the union between metabolic flexibility and personalized medicine. Clearly, there is still much work to be done, yet interesting times lie ahead.

## Abbreviations

ACC: acetyl-CoA carboxylase
AICAR: 5-aminoimidazole-4-carboxamide riboside
Akt/PKB: v-Akt murine thymoma viral oncogene homolog 1/protein kinase B
AMPK: adenosine monophosphate-activated protein kinase
BAT: brown adipose tissue
BCAA: branched chain amino acids
BCKD: branched-chain *α*-ketoacid dehydrogenase
CoA: coenzyme A
CPT-1: carnitine palmitoyltranferase-1
CR: caloric restriction
ER: endoplasm reticulum
FAO: fatty acid oxidation
FATP1: fatty acid transport protein 1
FGF21: fibroblast growth factor 21
FOXO: forkhead box protein O
GLP-1: glucagonlike peptide-1
GLUT: glucose transporter
HIF1*α*: hypoxia-inducible factor 1 alpha
HK: hexokinase
iNOS: inducible nitric oxide synthase
KB: ketone body
LAT1: large neutral amino acid antiporter
MEF2: myocyte-specific enhancer factor 2
mtDNA: mitochondrial DNA
mTOR: mechanistic target of rapamycin
mTORC1/2: mechanistic target of rapamycin complex 1/2
Myc: myelocytomatosis oncogene
NAD^+^: oxidized nicotinamide adenine dinucleotide
NADH: reduced nicotinamide adenine dinucleotide
NADPH: reduced nicotinamide adenine dinucleotide phosphate
NO: nitric oxide
NRF: nuclear respiratory factor
OXPHOS: oxidative phosphorylation
PDH: pyruvate dehydrogenase
PDK: pyruvate dehydrogenase kinase
PGC1*α*: peroxisome proliferator–activated receptor *γ* coactivator 1-*α*
PI3K: phosphatidylinositol 3-kinase
PPAR: peroxisome proliferator–activated receptor
PPP: pentose phosphate pathway
ROS: reactive oxygen species
SIRT: sirtuin
T2DM: type 2 diabetes mellitus
TCA: tricarboxylic acid
TFAM: mitochondrial transcription factor A
TFEB: helix-loop-helix leucine zipper transcription factor EB
WAT: white adipose tissue
